# Triboelectric and Piezoelectric Nanogenerators for Future Soft Robots and Machines

**DOI:** 10.1016/j.isci.2020.101682

**Published:** 2020-10-14

**Authors:** Min Pan, Chenggang Yuan, Xianrong Liang, Jun Zou, Yan Zhang, Chris Bowen

**Affiliations:** 1 Department of Mechanical Engineering, University of Bath, BA2 7AY Bath, UK; 2 National Engineering Research Centre of Novel Equipment for Polymer Processing, School of Mechanical and Automotive Engineering, South China University of Technology, Guangzhou 510640, China; 3 State Key Laboratory of Fluid Power and Mechatronic Systems, Zhejiang University, Hangzhou 310027, China; 4 State Key Laboratory of Powder Metallurgy, Central South University, Changsha, Hunan 410083 China

**Keywords:** Devices, Energy Systems, Nanostructure, Nanotechnology

## Abstract

The triboelectric nanogenerator (TENG) and piezoelectric nanogenerator (PENG) are two recently developed technologies for effective harvesting of ambient mechanical energy for the creation of self-powered systems. The advantages of TENGs and PENGs which include large open-circuit output voltage, low cost, ease of fabrication, and high conversion efficiency enable their application as new flexible sensors, wearable devices, soft robotics, and machines. This perspective provides an overview of the current state of the art in triboelectric and piezoelectric devices that are used as self-powered sensors and energy harvesters for soft robots and machines; hybrid approaches that combine the advantages of both mechanisms are also discussed. To improve system performance and efficiency, the potential of providing self-powered soft systems with a degree of multifunctionality is investigated. This includes optical sensing, transparency, self-healing, water resistance, photo-luminescence, or an ability to operate in hostile environments such as low temperature, high humidity, or high strain/stretch. Finally, areas for future research directions are identified.

## Introduction

Soft robots and machines can be considered as *soft systems* that aim to use highly compliant materials with elastic moduli that are comparable to soft biological materials and human tissues (kPa–MPa). These systems are of interest since they exhibit a high degree of mechanical flexibility and stretchability to replicate the properties of human skin and provide resilience. Highly compliant materials used in soft robots and machines are also often formed using low temperature processes, thereby providing ease of processing and low-cost fabrication. For soft systems to achieve their potential, the underpinning technologies of sensing, actuation, and the supply of power must be fully integrated and operate cooperatively.

Application areas for such soft systems include biomedical and medical devices, wearables, electronic skins (e-skins), and soft robots for human-machine interaction, which include assistive devices and rehabilitation training systems. There is also a need to create *end effectors,* which are devices that can be installed or attached to a robotic wrist or mounting plate to allow the robot to perform its intended tasks; these include manipulators, grippers, and robotic hands. Such soft effectors are desirable when there is a need to interact with fragile or soft components.

To create practical autonomous soft systems that are able to operate for long periods without electrical connection, the ability to operate while consuming minimal power is vital. Therefore, soft sensors should ideally be *self-powered*, in that they generate their own electrical signal in response to a stimulus. Likewise, soft actuators and effectors should have the potential to operate by harvesting ambient sources of energy, such as mechanical motion, strain, or heat. While there are a number of excellent reviews on soft robots (Bauer et al., 2014; Rus and Tolley, 2015; Cianchetti et al., 2018; George et al., 2018; Coyle et al., 2018; Chen et al., 2020a), triboelectric-/piezoelectric-based soft energy harvesters (Ding et al., 2019), e-skins (Dharmasena et al., 2019), wearable devices (Gunawardhana et al., 2020), and applications in health care (Chen et al., 2020), there is currently no review that has a focus on triboelectric- and piezoelectric-based soft devices for future soft robots and machines.

**Figure 1:**
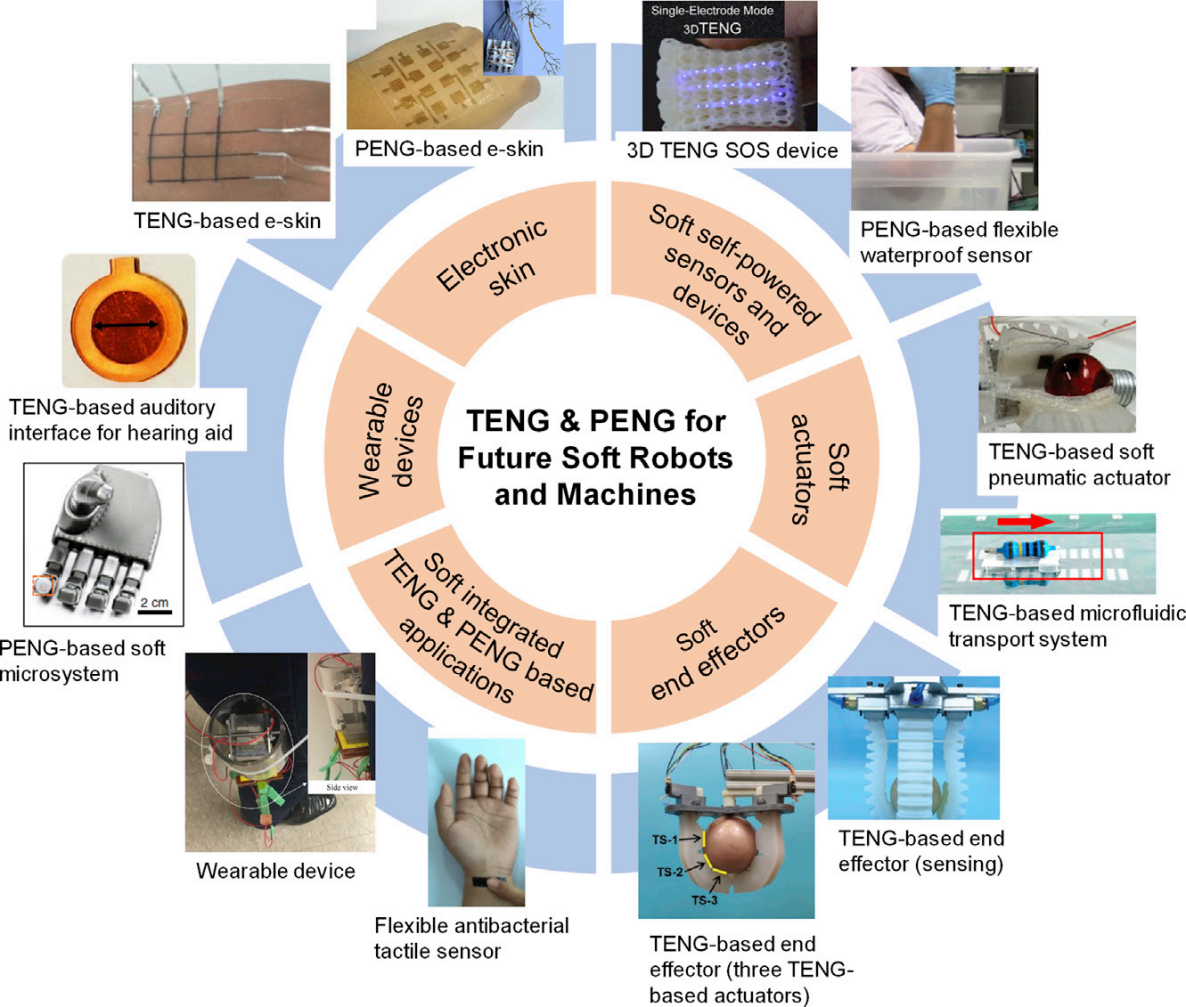
Current State-of-the-Art Triboelectric Nanogenerator (TENG) and Piezoelectric Nanogenerator (PENG) Technologies and Applications for Soft Robots and Machines

This perspective aims to overview the current state of the art in triboelectric and piezoelectric devices that are used as self-powered sensors and energy harvesters for soft robots and machines; hybrid approaches that combine the advantages of both mechanisms will also be explored, as shown in Figure 1. Efforts to improve system performance and efficiency by imparting a degree of multifunctionality to soft systems will also be discussed. This will include optical sensing, transparency, self-healing, water resistance, photo-luminescence (PL) or an ability to operate in hostile environments such as low temperature, high humidity, or high strain/stretch. Future directions for this intriguing research area will be explored.

## Mechanisms of Triboelectric and Piezoelectric Nanogenerators

**Figure 2:**
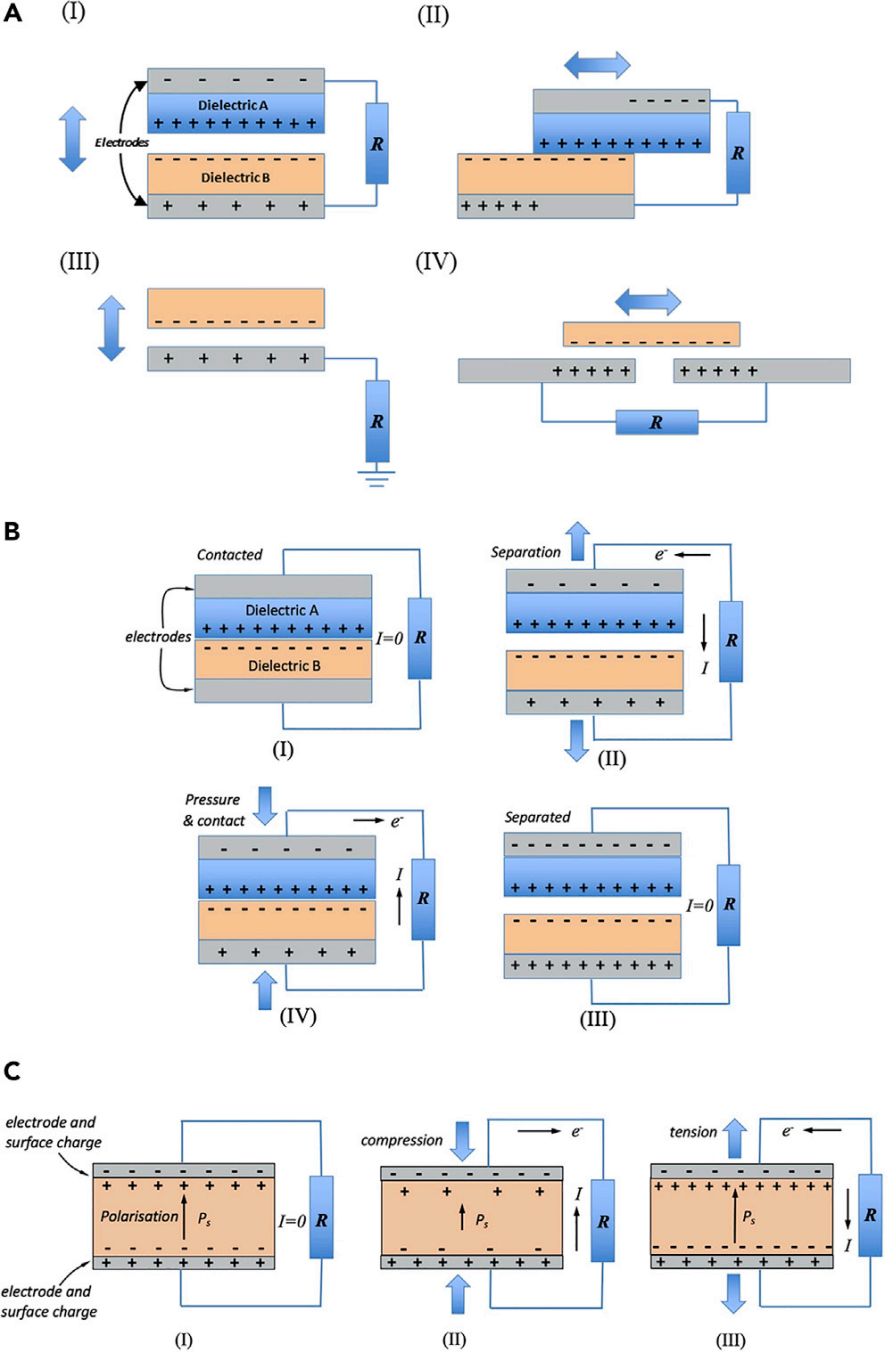
Fundamental Modes of Triboelectric Nanogenerators and Triboelectric and Piezoelectric Effects for Sensing and Harvesting (A) Four fundamental modes of triboelectric nanogenerators, (B) triboelectric effect in contact-separation mode for sensing and harvesting, (C) piezoelectric effect for sensing and harvesting.

It is of interest to initially consider the mechanisms by which triboelectric and piezoelectric charges are generated prior to discussion of their use in sensing and energy harvesting applications. Four fundamental modes have been developed for the triboelectric nanogenerator (TENG), which include the (i) vertical contact-separation (ii) in-plane sliding mode (iii) single-electrode mode and (iv) free-standing mode, as shown in Figure 2 (a) (Ma et al., 2018; Wang, 2017). The working principles of a TENG and its load matching behavior has been explained in detail using the parallel-plate capacitor model and the distance-dependent electric field model (Niu et al., 2013; Dharmasena et al., 2018; Niu and Wang, 2015; Elbanna et al., 2020). Each working mode exhibits its own structural characteristics, advantages, and limitations, and potential application area, which are compared and summarized in Table 1.

**Table 1:** Comparison of Four Fundamental Operational Modes of TENGs

Operational Modes	Structure	Advantages	Limitations	Key Parameters/Characteristics	Application areas and examples [Ref]
Vertical contact-separation	Vertical movement, large gap in between	High open-circuit output voltage	Pulse output	Average velocity, dielectric thickness; separation distance	Pressure, force, angle position, and pulse sensing (Hou et al., 2013; Luo et al., 2015; Ahmed et al., 2017; Mariello et al., 2020; Rasel et al., 2018; Zhao et al., 2019)
In-plane sliding	Horizontal/rotational movement, very small gap	High bandwidth, continuous and high electricity output	Poor long-term reliability	Sliding velocity and distance	Flow energy harvesting (Chen et al., 2018b; Yuan et al., 2020)
Single electrode	One operated electrode; one grounded	Simple, versatile, easy to fabricate; can be integrated on other devices	Relatively low output power;	Active aera size; electrode gap distance	Touching/typing screen (Wang et al., 2018a; Sun et al., 2018; Ren et al., 2018; Chen et al., 2016)
Free-standing	Multiple forms of movements, symmetric electrodes, asymmetric charge distribution	High energy conversion efficiency	Fixed electrodes; difficult to integrate	Free-standing height, electrode gap	Rotational and vibration energy harvesting (Nie et al., 2018)

TENG, triboelectric nanogenerator.

Figure 2B is a schematic of a *TENG* that is operating in a vertical contact-separation mode. Initially, in Figure 2B (I), the two layers of the device are pressed together by an external force to make *contact* and generate triboelectric charges at the interfaces of dielectric A and B. On release of the external force, the *separation* of the charged dielectric triboelectric layers leads to the generation of a potential difference between the two layers that can drive free charges in the electrodes to balance the potential. This leads to a current flow between the electrode when the device is connected to a load resistance (R), as in Figure 2B (II). Eventually, the surface charge is balanced and a current no longer flows, as in Figure 2B (III). When the films are pressed together again to make contact, the free charges that have accumulated at the electrodes can flow back into the circuit, which leads to a current flow in the opposite direction (Niu and Wang, 2015). Details of the materials and mechanism of the TENGs can be found in excellent recent reviews on typical applications in self-powered sensors (Hinchet et al., 2015; Wang et al., 2015), flexible power sources (Wang et al., 2017a), and wearable devices (Liu et al., 2019). New technologies, such as artificial intelligence, have also been integrated with nanogenerators. A comprehensive review about fabric-based PENGs and TENGs for artificial intelligence has been published by Dong et al. (2020), which can be read in parallel with this review.

The direct *piezoelectric effect* can also be used for sensing and harvesting. In this case, electric charge is generated as a result of a change in polarization due to the application of a mechanical stress. The current state of the art on nanostructured piezoelectric energy harvesters, including piezoelectric output from single-strained ZnO nanorods, the use of nanorod arrays, flexible substrates and alternative materials, and nanostructures has been reviewed by Briscoe et al. (2015). In Figure 2C (I) the piezoelectric material is in a stress-free state. If the material is polarized to a level, *P*_*s*_, such as in the case of a poled ferroelectric material, charge will collect at the material surface to maintain a charge balance. If a compressive stress is now applied to the material, as in Figure 2C (II), there will be a decrease in the level of polarization of the material and surface charge in now free to flow and generate an electric current in an external circuit. If the stress is removed, or a tensile stress is applied, the polarization level of the material will increase, leading to the flow of current in the opposite direction to maintain a charge balance.

In Figure 2, an electrical load resistance (*R*) has been connected to the triboelectric and piezoelectric sensor/generator. If the load resistance is infinite, the device is considered to be under *open-circuit* conditions, where there is no current and a maximum potential difference (*V*) is generated in response to the applied load; this condition can be of benefit to create high sensitivity voltage sensor. If the load resistance is reduced to zero, the device is under *closed-circuit* conditions, where there will be a maximum generated current (*I*) and no voltage. We will see in this review that both open-circuit voltage and short-circuit current are commonly reported for both triboelectric and piezoelectric devices. For energy harvesting applications, there is need to develop an optimum combination of voltage and current to generate power (*P*), since *P* = *VI*. Therefore, the load resistance needs to be optimized by impedance matching the load resistance to the energy harvester. Finally, it is of interest to note in Figure 2 that both triboelectric and piezoelectric generators generate an AC current since charges flow in both directions to balance surface charge; hence *rectification* of the output is needed for charge accumulation during energy harvesting.

In the following sections, we will review and discuss the current state-of-the-art TENG and PENG and integrated TENG and PENG-based soft robots and machines including their structure, materials, mechanism of operation, mechanical properties, and the output performance of the typical prototypes. These details are summarized in Table 2 for comparison.

**Table 2:** Summary of the Representative Triboelectric Nanogenerator- and Piezoelectric Nanogenerator-Based Soft Devices for Future Soft Robots and Machines

	Structure and Materials	Mechanism	Mechanical Properties	Sensing Performance	Output Performance	Additional Features/Applications	Reference
TENG	Laminated structure						
	PDMS/Cu + spacer (elastic sponge) + indium tin oxide (ITO)/polyethylene terephthalate (PET)	Contact-separation	/	/	Maximum short-circuit current 40 uA Maximum current density 0.8 μΑ/cm^2^ Maximum open-circuit voltage 220 V	Biomechanical energy harvesting	Hou et al. (2013)
	Polyimide (PI) + laser-induced graphene (LIG) + PTFE + PDMS	Contact-separation	Durability 40,000 cycles	/	Maximum open-circuit voltage 168V Maximum short circuit current density 21.3 mA/m^2^ Maximum power density 0.8 W/m^2^ (load resistance 20 MΩ)	/	Luo et al. (2015)
	Parylene-C + Au/Ti + PDMS + Kapton	Contact-separation	Young's modulus 1.549 MPa Tensile strength 5.154 MPa Strain at break 166.2%	/	Maximum peak-to-peak open-circuit voltage 1.6 V Maximum short-circuit current 0.15 μΑ Maximum power density 2.24 mW/m^2^ at 0.4 MΩ	Conformability and high charge-retaining capability	Mariello et al. (2020)
	PDMS + polyacrylamide containing lithium chloride (PAAm-LiCl) hydrogel + PDMS	Single electrode in contact-separation mode; In two-electrode contact-separation	Stretchability 1160% Transparency 96.2% Ultimate stress 446.2kPa at a stretch of 4.5 Durability 5000 cycles at single electrode mode and 20,000 cycles at two-electrode mode	Pressure Range 1.3–70 kPa Sensitivity 0.013 V/kPa	Maximum open-circuit voltage 145 V Maximum short-circuit current 1.5 μΑ Maximum Power density 35 mW/m^2^ at 70MΩ In two-electrode contact-separation mode: Maximum open-circuit voltage 182 V Maximum short-circuit current 20 μΑ	Ultra-stretchability; Transparency; Temperature range 0–60°C	Pu et al. (2017)
	Carbonized melamine sponge + PDMS (TENG sensor) or carbonized melamine sponge + multi-walled CNTs (strain sensor)	Contact-separation mode	Strain sensor: Durability 240,000 cycles	**Strain** Range 0–90% Maximum sensitivity 800% ((Δ*R*/*R*_0_)/*ε*)	TENG: Maximum open-circuit voltage 2 V Maximum short-circuit current 70 NA	Dual-mode and ultra-high sensitivity	Wang et al. (2017b)
	Silicone + PDMS + electrification liquid + ionic solution electrode	Liquid-solid contact electrification	Durability 50,000 times uniaxial tensile test Maximum stretchability 60%	/	Open-circuit voltage over 170 V (dry condition) over 10 V (liquid environment) Peak power density 18 mW/m^2^ (50 MΩ external load) 62.5 μW/m^2^ (300 MΩ external load)	Waterproof and underwater applications	Zou et al. (2019)
	Graphene films + TENG (polyethylene naphthalate (PEN) +PTEF)	Contact-separation mode	Durability 10,000 cycles	**Pressure** Range 0–100 kPa Sensitivity ((ΔI/I_0_)/ΔP) 1.63/kPa (0–6 kPa) 0.04/kPa (6–100kPa)	Average open-circuit output voltage ∼240 V Average short-circuit current ∼37μA Corresponding current density 9.3 μA/cm^2^	/	Chun et al. (2019)
	PET + Ag + PET/EVA + PDMS	Contact-separation mode or in-plane sliding mode	Durability 10,000 cycles	**Pressure** Range 1–150 kPa Sensitivity (d(ΔV/V_s_)/dP) 0.06/kPa (1–80 kPa)	/	/	Wang et al. (2016)
	PDMS + PI + Au	Contact-separation mode	/	**Displacement** Range 60–180° (6.9–1.2 pF) Sensitivity (*δ*(Δ*C*_*FD*_/*C*_*FD*0_)/*δ*θ) Up to 4.5/°	**Maximum voltage** 70 V **Peak current density** 2.7 μA/cm^2^ (5 MΩ load resistance)	/	Dhakar et al. (2016)
	Silicone rubber + AgNW + grating structured copper film + silicone rubber	Contact-separation mode	Durability 5800 cycles Stretchability 200%	**Pressure** Range 0-–-30 kPa Sensitivity 34 mV/Pa (<0.17kPa) 2.6 mV/Pa (0.17–1.7 kPa) 0.13 mV/Pa (>1.7 kPa)	/	/	Bu et al. (2018)
	Five layers: PET + ITO + PDMS + ITO + PET	Contact-separation mode	Thickness <500 μm	**Pressure** Response time 0.04 s Range 0.30-428.8 kPa Sensitivity 2.82 V/MPa	**Maximum open-circuit voltage** 1.61 V **Short-circuit current density** 47.31 mA/m^2^	Flexible, transparent and waterproof	Jiang et al. (2016)
	PDMS + poly(2-acrylamido-2-methyl-1-propanesulfonic acid) (PAMPS) ionogel + PDMS	Contact-separation mode	Stretchability Ultimate stress 125 kPa at a strain of 121% Durability 6000 cycles by a 0.4 N impulsive pressure at 1 Hz	**Force** Range 0.1–1 N Maximum sensitivity 1.76V/N	**Open-circuit voltage** 0.3 V (no stretch, 1 Hz of 1 N impulsive force) 3.3 V (50% strain, 1 Hz of 1 N impulsive force) **Maximum short-circuit current** 2.3 NA	Fully transparent	Zhao et al. (2019)
	VHB tape + poly(ionic liquid) (PIL) hydrogel + VHP tape + PIL hydrogel + VHP tape	Single electrode mode	Stretchability 710%	Capacitive mode: **Pressure** Range 1.1–45 kPa Sensitivity ((Δ*C*/*C*_0_)/Δ*P*) 0.57%/kPa Resistive mode: **Temperature** Range −20°C–60°C Sensitivity ((Δ*R*/*R*_0_)/Δ*T*) 11.3%/°C (-20–25°C) 2.1%/°C (25–60°C) **Strain** Range 0 to 160% Sensitivity ((Δ*R*/*R*_0_)/Δ*ε*) 2.7 Resistive mode with TENG: **Pressure** Range Up to 40 kPa Sensitivity (Δ*V*/Δ*P*) 1.8 V/kPa (<15 kPa) 0.13 V/kPa (15-50 kPa)	/	Anti-freezing and superior self-healing ability; Multiple functions of pressure, temperature, and strain sensing	Liu et al. (2020a)
	Polylactic-co-glycolicacid (PLGA) + Ag NW + PVA	Single electrode mode	Weight 80 mg Thickness 120 μm Stretchability 100% Durability 50,000 cycles at 40 N of 3 Hz	**Pressure** Range Up to 40 kPa Sensitivity ((Δ*V/V*_*s*_)/Δ*P*) 0.011/kPa	**Maximum power density** 130 mW/m^2^ (500 MΩ load resistance)	Breathable, biodegradable, antibacterial and all-fiber TENGs	Peng et al. (2020)
	TPU + PTFE + TPU + AgNW/TPU + VHB	Liquid-solid contact electrification	Thickness 300um Stretchability 200%; Durability 100,000 submerging-emerging cycles	/	**Open-circuit voltage** 120 V **Short-circuit current** 18 μA	Stretchable and shape-adaptive	Liang et al. (2020)
	PDMS + PTEF/AgNW + PDMS	Contact-separation mode; sliding mode	Durability 5000 cycles of 25 kPa at 0.5 Hz	**Pressure** Range 5–50 kPa Sensitivity 127.22 mV/kPa **Tangential sliding force (sliding mode)** Range 0.5–2 N	**Maximum open-circuit voltage** 3.14 V **Maximum short-circuit current** 26.29 NA **Maximum transferred Charge density** 23.98 μC/m^2^	/	Yao et al. (2020)
	Fluorinated ethylene propylene (FEP) + Cu + Kapton + Cu + air gap + PU + Cu	Sliding mode	Stretchability 200%	**Capacitive pressure sensing** Range 0–10 kPa Sensitivity ((Δ*C*/*C*_0_)/Δ*P*) 0.720/kPa (<1 kPa) **TENG sliding displacement sensing** Range 0–50 mm Sensitivity 0.1614 V/m, 53.92 nC/m	/	/	Yuan et al. (2020)
	Kapton + Al foil + DEA (carbon grease + elastomer)	Single-electrode structure in contact-separation mode Sliding mode (Chen et al., 2017b)	/	/	**Maximum open-circuit voltage** 1600 V (Chen et al., 2016) 3000 V (Chen et al., 2017d) **Maximum transferred charges** 350 nC (Chen et al., 2016) 390 nC (Chen et al., 2017d) TENG-DEA clamp (Chen et al., 2016) **Closing distance** 4 mm **Clamping force** 0.2 N	/	Chen et al. (2016) Chen et al., (2017b, 2017c, 2017d)
	Conductive sponge/porous silicone + skeleton (metal hinge) + silicone	Contact-separation mode	Bending for 20 min (3000 times) The resistance increases from 2.2 kΩ to 3.4 kΩ	**Force** Range 2.9–9.9 N (3.9–17 V) Sensitivity 1.8 V/N	**Maximum open-circuit voltage** 26 V **Maximum short-circuit current** 0.45 μA **Maximum transferred charge** 8 nC	Soft-rigid hybrid actuator	Chen et al. (2019)
	Stretchable electrode + PDMS + AgulisBlack/VeroWhite	Contact-separation mode	Maximum curvature 8.2 m^−1^	**Curvature** Range Up to 8.2 m^−1^ Sensitivity 0.0729 Vm^−1^ (<4.6 m^−1^) 1.0163 Vm^−1^ (4.6–8.2 m^−1^)	**Maximum open-circuit voltage** 24 V	Multi-material 3D printing	Zhu et al. (2020)
	TENG tactile sensor: silicone rubber + liquid metal (injected) + silicone rubber Curvature sensor: PDMS + liquid metal + PDMS	Contact-separation mode	/	/	**Maximum open-circuit voltage** 3 V	/	Yuan et al. (2019)
	Acrylic ring + Au + FEP + Au + Kapton	Contact-separation mode	/	**Sound** Range 50–110 dB Sensitivity 110 mV/dB (85–110 dB) Frequency response 100–5000 Hz	/	/	Guo et al. (2018)
	Polyimide + CNT-Nafion/Ionic membrane (IPMC) + Al	Free-standing mode	Durability 70% flexural rate after 10 cycles	/	**Maximum short-circuit current** 7.52 μA **Maximum transferred charge** 0.37 μC **Maximum power density** 10.88 mW/100cm^2^ (at 500 MΩ load resistance)	/	Yang et al. (2019)
	Vapor-responsive PDMS + Nylon + Al + Kapton + Al	Free-standing mode	/	/	**Maximum clamping force** 0.055 NA 80 cm^2^ Maximum clamping weight 6 g	/	Zheng et al. (2019)
	**Compliant tribo-skin** Silicone rubber + AgNW + silicone rubber **Inner TENG** PTFE + Cu + PET	**Compliant tribo-skin** Single electrode mode **Inner TENG** separation-contact mode	1000 contact-separation cycles in 16.6 min	**Compliant tribo-skin** **Force** Range 2–10 N Sensitivity 0.22N/V **Inner TENG** **Bending displacement** Range 10–150° Sensitivity 20°/V	**Compliant tribo-skin** **Average open-circuit voltage** 23 V **Short-circuit current** 50–60 NA **Transferred charge** 7.5 nC **Inner TENG** Maximum open-circuit voltage 8 V	Integration of two types of TENGs	Chen et al. (2020b)
	**Sandwiched structure**						
	Silicone rubber + carbon nanotubes + silicone rubber + urethane + carbon nanotubes urethane	Contact-separation mode	Stretchability 150% (FEM simulation) Durability 3000 pressing-releasing cycles	**Pressure** Range 0.25–32.1 kPa Sensitivity 1.52 mV/Pa (0.25–13 kPa) 1.073 mV/Pa (13–32.1 kPa) Response time 0.05s	**Maximum open-circuit voltage** 6.5 V **Maximum short-circuit current** 1.5 μA **Maximum transferred charge** 15 nC **Maximum output power** 0.3 μW	Stretchable self-powered and wireless keyboard	Ahmed et al. (2017)
	Healable PDMS + Ag nanowires and poly(3,4-ethylenedioxythiophene) (AgNW/PEDOT) film + Healable PDMS	Single electrode mode	Stretchability 50% Healing efficiency 100% Transmittance 73%	/	**Maximum power density** 327 mW/m^2^ (at 400 MΩ load resistance) **Maximum open-circuit voltage** 100 V **Maximum short-circuit current** 3 μA	Healing, stretchability transparency	Sun et al. (2018)
	Smearing carbon/silicone grease + dielectric elastomers	Contact-separation mode	Stretchability 100% Thickness 102um	/	**At contact area 9 cm**^**2**^ **Maximum open-circuit voltage** 115 V **Maximum short-circuit current** 3 μA	/	Chen et al. (2017a)
	PDMS + PMMA/PDMS + carbon black/PDMS	Single electrode mode	Stretchability 50%	**Force** Range 0.5–40 N Sensitivity 0.83 N/V (0.5–3 N) 2.50 N/V (3–40 N) **Pressure** Range 0.1–1.5 MPa Sensitivity 51.43 kPa/V	/	Multifunctional sensing	Ren et al. (2018)
	PDMS + carbon fibers + PDMS	Contact-separation mode	Continuous work of 10,000 s	**Pressure** Range 0.8–41.6 kPa Sensitivity 0.055 NA/kPa Response time 68 ms	Maximum power density 0.39 μW/m^2^ (at 100 MΩ load resistance)	High resolution of 127 × 127 dpi	Ma et al. (2017)
	**Encapsulation structure**						
	TENG for sliding sensing: CNT/PDMS Pressure sensing: CNT/PDMS of hybrid porous microstructure (HPMS) Energy supply: CNT/cotton fabric based porous supercapacitor (FPSC)	Sliding mode	/	**Sliding sensing** Peak to peak voltage 0.9 V **Pressure sensing** Maximum sensitivity (Δ*R*/*R*)/Δ*P* 35.7/kPa	/	Multifunctional sensing skin	Chen et al. (2018b)
	**3D Structure**						
	Resin + PAAm-LiCl hydrogel	Single electrode mode	/	/	**Maximum open-circuit voltage** 62 V **Maximum short-circuit volume current density** 26 mA/m^3^ **Peak power per unit volume** 10.98 W/m^3^ (at 0.75 TΩ load) **Transferred charge per unit volume** 0.65 mC/m^3^	3D TENG	Chen et al., 2018a
	Silicone wrap + Cu core	Contact-separation mode	Stretchability 20%	/	**Maximum open-circuit voltage** 5.75 V **Maximum short-circuit current** 0.38 μA **Maximum transferred charge** 0.65 nC **Maximum power density** 31.39 mW/m^2^	/	Tong et al. (2020)
	**Seesaw structure**						
	PDMS + aluminum + PDMS/laser-induced graphene (LIG)	Single electrode in contact-separation mode	/	**Velocity** 0.25–2.5 mm/s **Pressure** 0–400 kPa	/	/	Wang et al. (2018a)
	**Spring supported structure**						
	Au + PDMS + PDMS/CNT	Contact-separation mode	Stretchability 50%	**Pressure** Range 5–450 kPa Sensitivity 0.35 V/kPa (5-50 kPa) 0.51 V/kPa (50-100 kPa) 0.18 V/kPa (100–200 kPa) 0.04 V/kPa (200–450 kPa)	**Maximum peak power** 28 μW (at 2.5MΩ load resistance) **Energy conversion efficiency** 48% (at 2.5 MΩ internal impedance)	Impedance tunable; large-scale ultra-sensitivity	Rasel et al. (2018)
	Top part: PDMS/AgNw + Ti foam Bottom part: Ti foam + Al/Cu NW	Single electrode mode TENG (top part) Contact-separation mode TENG (bottom part)	/	**Force** Range 40–140N Sensitivity 28 mV/N	**Maximum open-circuit voltage** 90 V **Maximum short-circuit current** 9 μA	Dual-mode TENGs	Li et al. (2017)
	**Wrapped structure**						
	PET + Cu/PDMS + Al	Contact-separation mode	/	/	/	Roughly distinguishing the scale of objects	Li et al. (2019)
	**Sewing structure**						
	PI/Ag fiber + PTFE + Cu	Single-electrode mode	/	**Displacement** Range 0–180mm Maximum sensitivity 5.6 V/mm Resolution 1 mm	**Maximum open-circuit voltage** 55 V **Maximum short-circuit current** 118 NA	Non-contact motion identification	Wang et al. (2020a)
PENG	**Laminated structure**						
	PDMS + P(VDF-TrFE) + barium titanate (BT) NPs + PU	piezoelectricity	Durability 9000 cycles of stretching 30%	/	**Maximum Open circuit voltage** 9.3V **Maximum short circuit current** 189 NA (40% stretching)	/	Siddiqui et al. (2018)
	Fish swim bladder + Au	piezoelectricity	Durability 18,000 cycles up to 90 days	/	**Maximum Open circuit voltage** 10V **Maximum short circuit current** 51 NA (under 1.4 MPa compressive stress) 4.15 μW/cm^2^	/	Ghosh et al. (2016)
	PDMS + PMMA + Ti/Pt(or Ti/Au) + ZnO nano wires	piezoelectricity	Maximum compressive pressure 110 kPa	**Pressure sensitivity** 0.09 V/kPa	**Maximum output power** 35 μW (under 100 kPa) **Maximum open circuit voltage** 3V	/	Dahiya et al., 2018
	PET + 3D CdS nanowall array + NiO + cadmium foil	piezoelectricity	Bending degree 45°–120°	**Pressure sensitivity** 0.143 V/N	**Maximum open circuit voltage** 1.2V **Maximum short circuit current** 6 NA **Power density** 6.13 nW/cm^2^	/	Zhang et al. (2020a)
	Kapton + P(VDF-TrFE) micropillars + PDMS + Au	piezoelectricity	Maximum compressive force 2N	**Force** Range and sensitivity X axes: 0.02–0.44 N (0.3738 V/N) Y axes: 0.03–0.6 N (0.4146 V/N) Z-axes: 0.1–2 N (0.3443 V/N)	/	/	Chen et al. (2018d)
	ITO + PET + PDMS + BG-ZnO nanorods(random/aligned) + Ag NW-single walled carbon nanotube (SWCNT)	piezoelectricity	Minimum bending radius 31mm		**Maximum output voltage** 150 mV	/	Lee et al. (2017b)
	ITO + PVDF nanofibers	Single-electrode piezoelectricity	/	/	**Output voltage range** 40–60 mV	Transparent, cold/heat sensing	Wang et al. (2018b)
	P(VDF-TrFE) + ZnO nanowires	Piezoelectricity	Maximum temperature 55°C Maximum force 1.1 N	**Force** Range 0.4–1.4 N Sensitivity 2.1 V/N **Temperature** Range 328–383 K Sensitivity 0.18 mV/K	/	Pyroelectricity, “hot” and “pain” sensing	Sim et al. (2019)
	PDMS + P(VDF-TrFE) + Cu + sputtered Au	Piezoelectricity	/	**Force** Range <0.178 N Sensitivity 23 V/N	/	/	Lee et al. (2017a)
	**3D laminated structure**						
	PDMS + PVDF + Cr/Au	Piezoelectricity	/	**Force** Range 30 mN–3 N Sensitivity 60 mV/N	**Output Power frequency** 5–500 Hz	/	Han et al. (2019)
	**Kirigami structure**						
	P(VDF-TrFE) + BaTiO_3_ + Ag electrode	Piezoelectricity	Maximum normal force 60 N	/	**Maximum open circuit voltage** 6V **Maximum short circuit current density** 2 μA/cm^2^ **Output power density** 1.4 μW/cm^2^	All 3D-printed structure	Zhou et al. (2020)
	**Laminated structure**						
Integrated TENG and PENG	Ag NW@PTFE + PVDF + graphene	Triboelectricity, piezoelectricity and pyroelectricity	Elastic modulus 630 MPa Breaking elongation 9.1% Durability: 10,000 stretching cycles	**Pressure** Range 0.1–2.1 KPa sensitivity 0.092 V/KPa **Temperature** Range 10–45°C Sensitivity 0.11 V/°C	/	Antibacterial	Ma et al. (2019a)
	PDMS + PCB + Cu	Piezoelectric and triboelectric	/	/	**Maximum open circuit voltage** 1.2 V **Maximum short circuit current** 30 NA	Machine learning	Fang et al. (2020)
	PDMS + Cellulose/BaTiO_3_	Piezoelectric and triboelectric	/	/	**Maximum output voltage** 48 V **Maximum output power** 85 μW	/	Shi et al. (2019)
	**Woven structure**						
	PDMS substrate + hemi-spherical PDMS + CNT + Ag NWs + (3-Aminopropyl) triethoxysilane (APTES) + tridecafluoro-(1,1,2,2-tetrahydrooctyl)-1-trichlorosilane (FOTS)	Capacitive and piezoresistive sensors Triboelectric and piezoelectric energy harvester	/	Capacitive mode: **Force** Range 0–0.8 N Sensitivity 0.627 pF/N Piezoresistive mode: **Displacement** Range 0–10 mm (5.3–7.2 Ω)	**Power density** 422 mW/m^2^	/	Kim et al. (2019)
	**Wave structure**						
	Silicone gel + Ag + PVDF	Triboelectric and piezoelectric principles	Bending angle range 30–90° Maximum stretching strain 30%	/	**Maximum output power** 490 nW	/	Liu et al. (2020b)
	**Spring-mass system**						
	Nylon + PTFE	Triboelectric and piezoelectric principles	/	/	**Output voltage** under 1.0 g excitation 50 V (PENG) 60 V (TENG)	/	Li et al. (2018)

## Applications of TENGs

The application of TENGs to self-powered sensors, electronic skins, soft actuators, and effectors will now be described.

### Energy Harvesting for Self-Powered Soft Systems and Sensors

#### Energy Harvesting for Self-Powered Soft Systems

Soft self-powered systems are in high demand for a wide range of applications, which include wearable, biomedical devices, and self-powered and intelligent robotic systems. TENGs are able to efficiently convert ambient forms of energy, such as the kinetic energy of water droplets or waves or biomechanical and mechanical motion, into electricity through its energy harvesting mechanism. These devices have significantly contributed to the creation of self-powered systems, where TENGs have provided opportunities that range from the delivery of power to small electronics (μW to mW) or contributing to large-scale energy harvesting through engineering design (W to kW). The charge generated by the TENG can be used as a sensor, which requires not external power source, and can be considered as a *self-powered sensor*, which will also be described. Innovative self-powered prototypes and devices have been successfully developed in the recent decade, which include TENG-based soft self-powered micro-supercapacitor power supplies, sensors in automotive applications, and wearable mechano-sensors for organ and health monitoring.

Hou et al. (2013) developed a cost-effective and easy to fabricate TENG based on contact-separation between a polydimethylsiloxane (PDMS) film and a polyethylene terephthalate (PET) film to harvest human walking energy. An elastic sponge was used as a spacer between the indium tin oxide (ITO)/PET, and PDMS/Cu layers in the TENG, where the optimal spacer thickness was 3mm and a TENG with an area of 4.5 × 4.5 cm^2^ was able to generate a short-circuit current density up to 0.8 μΑ/cm^2^ and an open circuit voltage of 135 V. A TENG-based shoe insole was developed which can provide a maximum output voltage of 220V and a current density of 40 uA/cm^2^. Using a laser engraving technique to effectively integrate a graphene-based TENG with a micro-supercapacitor array into a single device, Luo et al. (2015) fabricated a flexible, self-charging, and cost-effective micro-supercapacitor power unit. A high degree of integration was realized through the double-faced laser engraving of a polyimide (PI) substrate. The two sides of the laser-induced graphene (LIG) electrodes were used separately for fabricating the TENG and micro-supercapacitor array. The LIG-TENG exhibited a peak power density of 0.8 W/m^2^ at a load resistance of 20 MΩ, and the micro-supercapacitor had a high capacitance of approximately 10.29 mF/cm^2^ at a current density of 0.01 mA/cm^2^. The micro-supercapacitors could be charged by harvesting mechanical motion energy, and this was demonstrated by continuously power light-emitting diodes and a commercial hygrothermograph.

TENGs have also shown promising potential for self-powered soft human-machine interaction devices. Ahmed et al. (2017) developed a flexible, self-powered wireless keyboard based on vertical contact-separation mode TENG, which was able to elastically stretch to a maximum strain of ~150%. The keyboard was fabricated using urethane, silicone rubber, and carbon nanotubes (CNTs) electrodes. The keyboard was able to convert the mechanical energy of finger tapping during regular typing into electrical energy based on contact tribo-electrification, which can eliminate the need of external power source. It was shown that after 90s of regular typing on a key, it was possible to charge a 10 *μF* capacitor up to 2.5 V, with the maximum power of 0.3 *μW*. Mariello et al. (2020) developed a novel flexible single-electrode TENG based on a combination of a polysiloxane elastomer and a poly (para-xylylene). The TENG operated using a contact-separation mode, as in Figure 2A, for low-frequency energy harvesting of intermittent tapping loads, such as finger or hand tapping. The devices can be used for driving low power consumption soft wearable devices.

**Figure 3:**
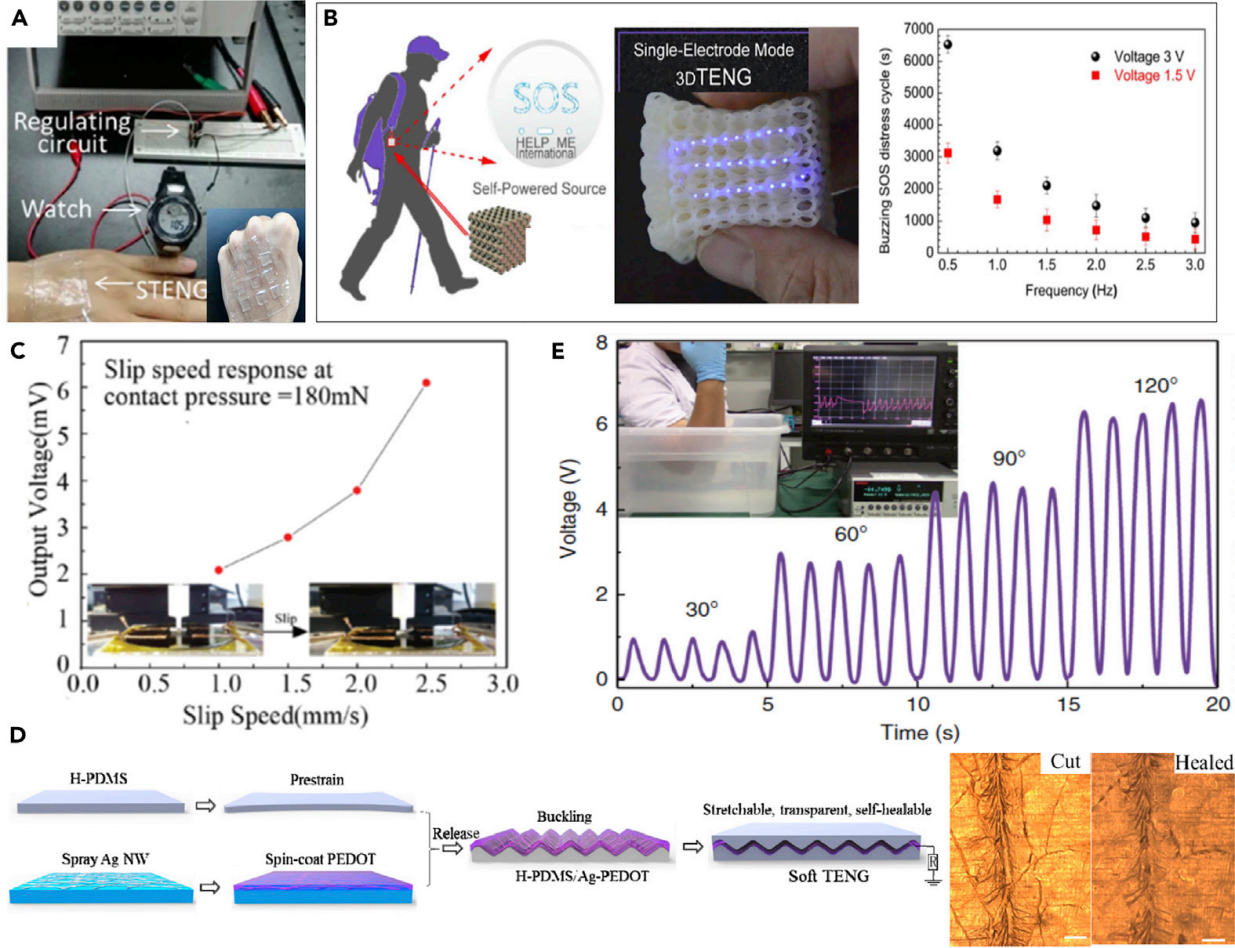
TENG-based Soft Self-Powered Sensors and Devices for Energy Harvesting (A) TENG-based energy harvesting watch and soft skin-like TENG sensor. (B) flexible, self-powered SOS device based on a 3D-printed TENG. (C) TENG-based tactile sensors for slip detection. (D) soft TENG-based self-healing and self-powered energy skin. (E) waterproof wearable TENG devices for underwater applications.

A soft skin-like TENG was developed by Pu et al. that enabled both biomechanical energy harvesting and tactile sensing using a combination of a hybridizing elastomer and ionic hydrogel as the electrification layer and electrode (Pu et al., 2017). In this case, a sandwich-like architecture was used for design of the soft TENG, as shown in Figure 3A. A polyacrylamide (PAAm) hydrogel containing lithium chloride (LiCl) was used as the ionic hydrogel (PAAm-LiCl hydrogel), and commonly used PDMS were adopted as the elastomer layer. The soft TENG could provide electrical energy with an instantaneous peak power density of 35 mW/m^2^ to supply wearable electronics with energy converted from human motion. For the application of an energy-harvesting watch, a combination of ultra-high stretchability (uniaxial strain, 1160%) and transparency (average transmittance, 96.2% for visible light) could be achieved simultaneously. The soft TENG device was also pressure-sensitive and able to sense pressures as low as 1.3 kPa for application as an artificial electronic skin for touch/pressure perception, and self-powered soft devices. Similarly, by harvesting biomechanical energy, Chen et al. (2018a) developed an ultra-flexible, biocompatible, and structure adjustable three-dimensional TENG (3D-TENG). The 3D-TENG was fabricated using 3D-printed composite resin parts, with a printing precision of 1 um, and an ionic hydrogel as the electrification layer and electrode. An emergency SOS self-powered device was designed based on the 3D-TENG by harvesting biomechanical energy. A sustainable output power of 10.98 W/m^3^ (peak power per unit volume) and transferred charge per unit volume of 0.65 mC/m^3^ were produced during a low triggering frequency of ~1.3 Hz, as shown in Figure 3B. It is suggested that water evaporation of the hydrogel has a potential risk of weakening the degree of triboelectrification due to a decrease in surface charge density if the 3D-TENG is not well sealed from the environment. Wang et al. (2017b) developed a miniaturized all-in-one TENG with high structural durability, anti-impact, and multi-mode harvesting of energy from compression, swaying, and magnetic fields. The device was developed by assembling shear thickening fluid and magneto-sensitive films. The TENG was able to provide a maximum power density of 27.05 mW/m^2^ with a voltage of 10.40 V at 10 MΩ under compression. The device with shear thickening fluid exhibited high anti-impact properties and could resist and dissipate harsh collision forces ranging from 409 N to 1390 N, demonstrating excellent safeguarding properties of TENG for human wearers. A wearable smart glove was developed based on the all-in-one TENG for precisely mapping finger joints motion.

### TENG-Based Self-Powered Sensors

Tactile sensors for slip detection are essential for human-like steady control of a dexterous robot hand. Wang et al. (2018a) proposed a flexible slip sensor based on TENG with a seesaw structure. The sensor consists of two porous PDMS layers separated by an inverted trapezoid structure with a height of 500 μm. Two independent TENGs that operated in a single-electrode mode within a single unit were designed to detect slip from different directions. LIG was arranged in the bottom PDMS layer to act as the induction electrode. The output voltage of the sensors increased from 2 mV to 6 mV, when the slip velocity increased from 1.0 mm/s to 2.5 mm/s, as shown in Figure 3C. A linear relationship between the pressure and resistance change was also achieved. The sensor was also able to detect slip and static normal forces by using a piezoresistive LIG electrode, thereby combining signals from two sensing mechanisms. Wang et al. (2017b) also exploited this hybrid approach to develop a dual-mode sensor derived from high temperature carbonized melamine sponge that acts as both a TENG sensor and piezoresistive sensor for monitoring human activities. In the self-powered TENG sensory mode, the sensor generated an average open circuit voltage up to ~2 V and short circuit current up to ~70 nA when being used as self-powered triboelectric sensor, which was sufficiently sensitive for detecting finger touching and plantar pressure distribution of human feet. It was found that the incorporation of multi-walled CNTs into the sponge greatly enhances the sensitivity of original carbon-sponge when used as piezoresistive strain sensor. In the piezoresistive sensory mode, by incorporating CNT into the sponge, the sensitivity of the sensor greatly improved up to nearly 800%, reflected by the relative change in current or resistance. However, when used in piezoresistive more electrical energy is needed to determine the change in resistance with strain. In addition, the cyclic stability of the sensor material is highly improved by addition of CNTs due to the mechanical reinforcement of the sponge fibers and additional conductive network provided by nanotube network. Zhang et al. (2020b) developed a flexible, transparent, and self-powered UV photodetector by coupling TENG and photoelectric effects. The device integrated a flexible ZnO nanoparticle (NP) UV photodetector, a transparent and flexible film-based TENG (TFF-TENG), commercial chip resistors, and LEDs on a PET thin film. The TFF-TENG could harvest mechanical energy from finger tapping and sliding motion and power the ZnO NP UV photodetector to realize self-powered detection. The voltage of the constant resistors connected with the UV photodetector in series changed from 0.5 to 19 V when illuminated with UV light, with power intensities increasing from 0.46 to 21.8 mW/cm^2^; the voltage variation is reflected by the number of LEDs.

For large-scale, ultra-sensitive pressure sensing applications, Rasel et al. (2018) developed a highly efficient TENG by detecting and harvesting pressures ranging from 5 kPa to 450 kPa, with a high sensitivity of 0.51 V/kPa. An estimated energy conversion efficiency of 48% was achieved by optimizing and tuning the internal impedance of the TENG at 2.5 MΩ. The TENG was based on double-side tribological layers of micro-patterned PDMS and PDMS-multiwall CNT nanocomposites. The PDMS and PDMS-CNT nanocomposite layers were micro-patterned via sandpaper replication to increase the effective contact area in order to enhance the electrical output of the triboelectric self-powered and maintenance-free sensing devices. Potential applications of the soft sensors include sport science, high-risk diabetic foot ulceration, and rehabilitation. To enhance the capability of soft power devices, Sun et al. (2018) developed a self-healable, stretchable, transparent, and energy harvesting TENG as a soft power source which can provide a high voltage of ~100 V and a maximum power density of 327 mW/m^2^. The single-mode TENG was based on a buckled conductive thin electrode that was sandwiched between two healable PDMS (H-PDMS) films, as shown in Figure 3D. The H-PDMS could heal the damaged Ag nanowires and poly(3,4-ethylenedioxythiophene) film to restore its electrical conductivity to recover its ability for energy-generation after accidental damage such as cuts, with a 94% healing efficiency. A soft TENG-based self-healable energy skin was also developed, showing good conversion of biomechanical energy into electricity (~100 V, 327 mW/m^2^), which can be used for soft wearable devices, biomedical and health-care applications.

The development of a waterproof, long-term, and sustainable power source is needed for soft wearable electronics for underwater applications. Zou et al. (2019) developed a bionic stretchable nanogenerator for underwater energy harvesting that mimics the structure of ion channels on the cytomembrane of electrocyte in an electric eel. The design is flexible, stretchable, fatigue resistant and can be used underwater. Inspired by an electric eel, a mechanical control channel was manufactured by exploiting the stress-mismatch between PDMS and silicone to mimic the structure of ion channels on the cytomembrane of an electrocyte in an electric eel, see Figure 3E. The TENG devices could achieve an open-circuit voltage of over 170 V in dry conditions and over 10 V in a liquid environment, which can be used for energy harvesting and underwater sensing. In single electrode mode, the peak power density of the device can reach 18 mW/m^2^ with an external load resistance of 50 MΩ, while in liquid-solid contact mode, the peak power density can reach 62.5 μW/m^2^ with an external load of 300 MΩ. The soft system was worn on the arthrosis of a swimmer for motoring human body multi-position motion during swimming using wireless transmission. The results suggested a miniaturized device has the potential to act as a body mechanical energy harvester or sensor for implantable applications, such as harvesting heart beating energy and sensing pulse signals, and soft robots. To enhance the usability of the TENG-based power source in extreme environments, Bao et al. (2020) developed an anti-freezing hydrogel-based TENG (AH-TENG) as a supply source for wearable devices. The device was able to harvest human biomechanical energy in extreme low-temperature environments, as low as −69°C. A 3 × 3 cm^2^ AH-TENG was able to provide an output voltage of 285 V and an instantaneous peak power density of 626 mW/m^2^. By integrating a TENG with other energy harvesting techniques, Wei et al. (2020) developed a hybridized mechanical and solar energy-driven self-powered hydrogen production system. They exploited a rotatory disc shaped TENG to harvest mechanical energy from water flow wave to function as an external power source and couple to a WO_3_/BiVO_4_ heterojunction photoanode to form a photo-electro-chemical water splitting cell to produce H_2_. After transformation and rectification, the peak current reached 0.1 mA at the rotation speed of 60 rpm. Such a TENG-based self-powered system can be used to facilitate photo-electro-chemical hydrogen generation.

Three-dimensional printing and machine learning technologies have been exploiting for fabricating TENGs to enable high precision and cost-efficient fabrication. Tong et al. (2020) reported on a novel 3D printing method using elastomeric metal-core TENG fibers. The TENG could be directly printed in the form of stretchable membranes, meshes, and hollow 3D structures on planar, rotating, and non-planar anatomical substrates. The utility of the flexible silicone-copper TENG fibers and 3D printing process were demonstrated by creating wearable mechano-sensors for organ and human activity monitoring. In combination with machine-learning signal processing algorithms, the mechanosensors were able to measure perfusion-induced kidney edema and speech recognition, with a 99%-word classification accuracy. The work demonstrates the potential of 3D-printed triboelectric devices for self-powered sensing applications.

Other forms of energy harvesting technologies such as solar cells, thermoelectrics, electromagnetic generators, and flexible batteries are being development for future soft robots and machines. New manufacturing and fabricating approaches are being developed to create *soft* and *flexible* varieties of such energy technologies. Solid metal coils are currently used in conventional electromagnetic actuators which could be difficult to integrate with soft robots or machines and can be large and bulky. New soft electromagnetic actuators have been developed which use liquid-metal channels embedded in elastomeric shells (Mao et al., 2020). Such devices could overcome this barrier and provide a new stretchable, durable, and programmable actuation technology for soft applications. The maximum power density of this new form of electromagnetic actuator is approximately 5.3 mW/g, which can be used to power small soft devices such as micro-soft robots for drug deliver applications. The mechanism of electromagnetic generator is resistive-free electron conduction driven by Lorentz force, while that of TENG is a capacitive displacement current due to the polarization of surface electrostatic charges. The output advantages of a TENG over electromagnetic generator, particularly at low frequencies, provide a wider range of applications for TENGs in energy harvesting, such as application in water wave energy harvesting for underwater soft robots.

Flexible batteries are also being developed rapidly along with wearable electronics. They are predominantly based on Zn-MnO^2^, Zn-Ag, LIBs, ALBs, Li-air, Al-air based systems which exhibit high stretchability and good capacity. However, the energy density of newly developed flexible batteries is between 0.53 mWh/cm^2^ to 8.14 mWh/cm^2^ (Song et al., 2019), which may not be sufficient to continuously power soft robots and machines. In addition, there is a trade-off between the energy density and stretchability, while high energy density generally results in low stretchability of the batteries, which could restrict the design of future soft robots and machines. TENGs have the advantages of high output power and can continuously power soft robots through the energy harvesting mechanism. Recently, flexible lithium-ion battery has been integrated with a fabric-based TENG to form wearable electronics (Pu et al., 2015). Solar energy has also been integrated with TENGs for the development of flexible self-charged devices (Ma et al., 2019b), and solar-based flexible electrodes for e-skins (Wangatia et al., 2020) can be implemented on soft machines, such as biomedical robots. A solar cell can be also combined with flexible batteries to provide a compact continuous power solution for soft actuators and robots (Wehner et al., 2014). The integration of the state-of-the-art energy technologies with TENG provides new direction for the design of untethered soft robots, which is currently facing a challenge caused by the restriction of power supplies.

### TENG-Based Electronic Skins for Soft Robotic Systems

**Figure 4:**
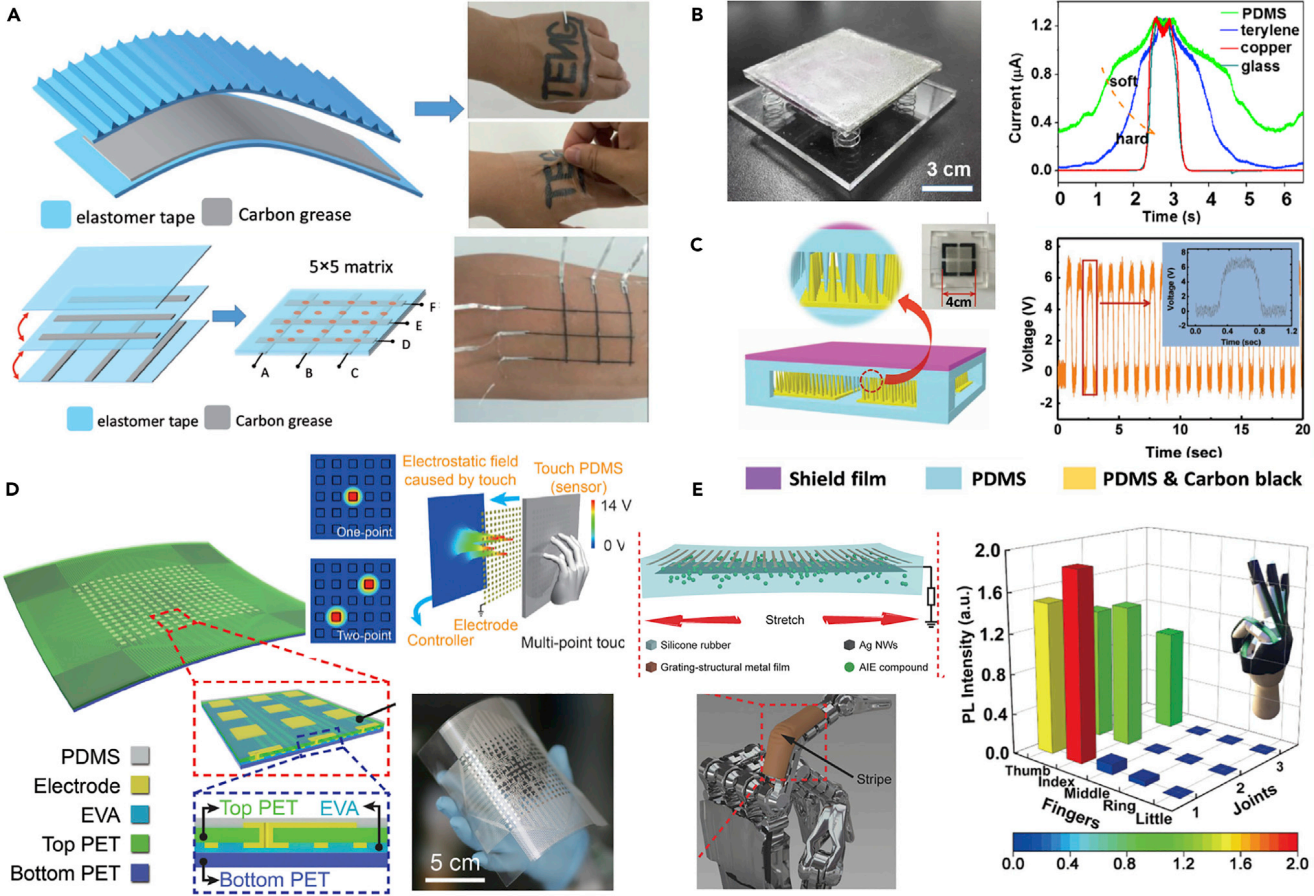
Newly Developed TENG-based Electronic Skins (e-Skins) for Soft Robotic Systems (A) ultrathin (102 um), high stretchable (100%) e-skins for tactile sensing. (B) smart tactile e-skin that can “feel” the hardness of the contact material by detecting the change of current peak. (C) TENG-based fully elastic and metal-free tactile e-skin for detecting normal and tangential forces, and its demonstration by an output voltage for vibration measurement, yellow region is PDMS and PDMS carbon black, pink is shield film. (D) flexible, highly sensitive 16 × 16 pixelated triboelectric sensor matrix that can detect single-point and multi-point tactile stimuli. (E) stretchable triboelectric-photonic smart skin that demonstrated a multidimensional tactile and gesture sensing for a robotic hand; a 3D normalized photo-luminescence intensity map of a hand gesture “OK”.

Electronic skins (e-skins) have been developed to mimic the functionalities of human, or animal, skin so that they are able to sense environmental factors such as touch, heat, cold, force, and pressure (Someya and Amagai, 2019). Soft TENGs can be stretchable, ultrathin, highly sensitive, self-powered, and conformal to human body to allow the capture of signals from subtle body motions and external stimuli, which can be used to fabricate electronic skins. Investigations into the development of TENG-based e-skins are progressing for human machine interfaces, soft robotics, and health-care monitoring systems. Novel designs of TENG-based electronic skins has been developed recently and Chen et al. (2017a), who proposed a TENG with an ultrathin thickness (102 um) and high stretchability (100%) that was able to act as a second skin on the human body, without impairing body movement. The TENG was fabricated by smearing carbon/silicone grease onto the surface of a dielectric elastomer, and the carbon grease when then sandwiched between dielectric elastomers to form a stretchable electrode, as shown in Figure 4A. A fine control of the electrode geometry was achieved to adaptive design of different arrangements of sensing skins for a range of application. An electrode network sensory matrix and a multilayer structure sensory system were created to detect tactile touching with an output voltage ~ 4V, see lower image of Figure 4A.

Li et al. (2017) developed a smart tactile e-skin that was able to “feel” the hardness of the contact material by quantifying the shape change of the current peak produced by the triboelectric effect, as shown in Figure 4B. The tactile sensor provides simultaneous detection of contact, hardness, and location by detecting the current peak. The sensor was able to detect a linear pressure that ranged from 40 to 140 N, with a high sensitivity up to 28 mV N^−1^. For force measurement applications, Ren et al. (2018) proposed a fully elastic and metal-free tactile e-skin that can detect both normal and tangential forces. The TENG, operating in single-electrode mode, was employed as the core element for signal generation. An elastic film made from PDMS was used as the dielectric material for triboelectrification, and the stretchable electrodes are made by mixing PDMS and carbon black (PDMS-CB), see Figure 4C. For force measurement, a wide range of tangential force (0.5–40 N) could be accurately measured with a sensitivity of 0.83 N V^−1^ (0.5–3 N) and 2.50 N V^−1^ (3–40 N). For pressure measurement, the measured pressure can be 1.5 MPa, with a sensitivity of ~51.43 kPa V^−1^. The sensor was able measure the tangential force from different directions and the twisting force based on the four-partitioned electrodes structure. The sensor was applied to tactile sensing and mechanical vibration detection, with typical output voltages shown in Figure 4C. The sensor has a potential in wearable electronics and for human-machine interactions.

Chun et al. (2019) developed a self-powered flexible neural tactile sensor that imitates the function of human finger skin. The tactile sensor can be sensitively activated using pressure or vibration stimuli using independent laminated sensor elements. To mimic the slow adaptive mechano-receptors of human skin, a sensor array consisting of a high-density of interlocked percolative graphene films was fabricated to detect pressure based on piezoresistance; defined as a change is resistance with stress or strain. The TENG was laminated on the sensor array to detect high-frequency vibrations and mimic the fast-adaptive response of mechano-receptors, and produce electric power. The output voltages of the neural tactile sensor were strikingly similar to the real neural spike signals of human skin, and the sensor was able to recognize the different textures of 12 fabrics.

To improve the resolution and sensitivity of TENG-based e-skins, Wang et al. (2016) designed a flexible 16 × 16 pixelated triboelectric sensor matrix that can map single-point and multi-point tactile stimuli in real time via multichannel data acquisition while being able to maintain an excellent pressure sensitivity and long-term durability. The skin structure is shown in Figure 4D, where a square piece of PET film with a thickness of 250 μm was utilized as the flexible dielectric substrate. The electrodes consisted of two parts: an aligned regular electrode array on the upper side to serve as the charge-sensing element and circuit configuration electrodes on the lower side that are connected to external measuring equipment. To enhance the triboelectric effect, the cured PDMS layer was dry etched to produce a micro/nanostructured surface to increase the effective charge density. The e-skin was demonstrated for the real-time single-point and multi-point touching detection. To simplify the fabrication process of TENG-based e-skin, Ma et al. (2017) developed an easy-to-fabricate cost-efficient self-powered e-skin with high resolution. The e-skins were fabricated based on a sandwich structure, where the upper and lower PDMS layers act as a contact surface in terms of its biocompatibility, high flexibility and low weight. Carbon fibers were integrated onto the PDMS layers to act as the electrically conductive electrode. The two groups of carbon fibers were electrically insulated and placed perpendicular to each other. The resolution of the e-skin increased with an increase in carbon fiber fraction and an e-skin with an area 0.3 cm × 1.0 cm was attached to a finger to demonstrate its flexibility and stretchability, where a periodic current was detected when a periodic pressure was applied to the e-skin using a steel tip.

To measure the static position and dynamic motion of human fingers, Dhakar et al. (2016) developed an e-skin using PI (Kapton) as the substrate due to its excellent adhesion properties with gold, compared to other flexible materials such as PDMS, and its superior mechanical properties. PDMS films with micropatterned structures were bonded on one side of a PI substrate, and a 50 nm gold film was coated on the other side to act as an electrode. When measuring the angle of joints at different positions of a finger, the sensor was able to generate a peak voltage of 70 V and a peak current density of 2.7 μA/cm^2^ at a load resistance of 5 MΩ, which is suitable for application in intelligent wearable devices. To improve the multi-dimensional measurement capability for TENG-based e-skins, Bu et al. (2018) developed a stretchable triboelectric-photonic smart skin that demonstrated a multifunctional tactile and gesture sensor for a robotic hand. This skin was composed of a soft aggregation-induced photoemission active substrate with a tightly bonded microcracked copper film and a middle stretchable conductive layer based on a silver nanowire network. Multi-dimensional tactile and gesture sensing was achieved by coupling PL, which is light emission after the absorption of photons, and triboelectrification. The triboelectric-photonic smart skin exhibited a tunable-induced emission in response to lateral tensile strains in the range of 0–160%, and it can be used as a TENG for vertical pressure sensing with a maximum sensitivity of 34 mV Pa^−1^. The device has been demonstrated on a robotic hand and a 3D normalized PL intensity map of a hand gesture “OK” is shown in Figure 4E.

**Figure 5:**
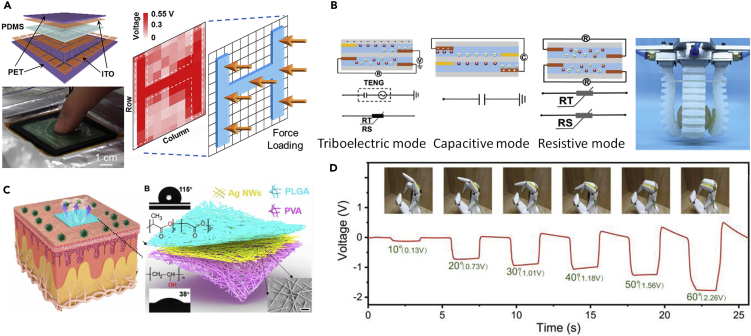
Enhanced TENG-based Electronic Skins (e-Skins) for Soft Robotic Systems with Transparency, Waterproof, Self-Heal, and Biodegradability (A) integrated triboelectric tactile sensor array with flexible, transparent, and waterproof features. (B) PENG-based self-healing e-skin based on zwitterionic poly (ionic liquid) (PIL) with a soft gripper demonstration; the ionic skin has capacitive, resistive and triboelectric sensing modes. (C) breathable, biodegradable, and antibacterial e-skin based on all-nanofiber TENG with micro-to-nano hierarchical porous structure, which can be attached to epidermis. (D) bioinspired ultrathin self-powered TENG-based e-skin with enhanced triboelectric effects and sensitivity; demonstration of using e-skin on a robotic hand for pressure measurement at a variety of bending degrees.

Transparency, waterproofing, self-healing, and biodegradability for advanced e-skins is attracting increasing interest and TENG-based enhanced e-skins have been developed to include these functionalities. Jiang et al. (2016) developed an integrated triboelectric tactile sensor array with flexible, transparent, and waterproof features. This waterproof tactile sensor array consisted of five layers of materials from top to bottom, including a polyester (PET) protective layer, a conductive ITO layer, a PDMS layer, an addressable ITO electrode layer, and a supporting PET layer; the device structure is seen in Figure 5A. This TENG-based e-skin was able to generate an open circuit voltage up to 1.61 V and a short circuit current density of 47.31 mA/m^2^. In a tactile panel device, a 10 × 10 array of PDMS micropyramids were separated by PDMS side walls. Two-dimensional touch mapping was demonstrated, as shown in Figure 3A, with an H-shaped PDMS mold pressed on the sensor array to demonstrate its sensing capability. The average voltage peak of the touched pixels is 0.62 ± 0.18 V, corresponding to a pressure of 823.1 ± 234.3 kPa, while the average peak of the unloaded pixels is 0.17 ± 0.08 V. Zhao et al. (2019) reported on a fully transparent, highly stretchable, and self-powered contact-separation TENG that acted as a tactile sensor. This TENG design consists of a double network ionogel with a high transparency, stretchability, and conductivity to act as the electrode and friction layer, and patterned PDMS as another friction layer. Two aluminum belts were attached to the two ionogel films for electrical connection, respectively. The fabricated sensor exhibited a maximum sensitivity of 1.76 V N^−1^ when detecting impacting forces in the range of 0.1–1 N. The soft sensor had good stretchability and the triboelectric signals maintained a good linearity with impacting forces at a range of tensile strain ratios (0%, 10%, 50%, and 80% strain). The sensor was used for detecting multiple forms of motion, including stretching, twisting, touching, finger bending, airflow, and human wrist pulse.

A self-healing feature has been investigated and equipped for TENG-based e-skins to enhance their damage resistance. Liu et al. (2020a) used a zwitterionic poly (ionic liquid) for preparing hydrogels to fabricate an e-skin with super-stretchability (~900%), self-healing ability, and high electrical conductivity (1.1 S m^−1^), even at low temperatures of −20°C. The poly (ionic liquid) gel was prepared via free-radical copolymerization of a zwitterionic IL monomer, 1-vinyl-3-(carboxymethyl)-imidazolium, and acrylamide (AAm) in KCl aqueous solution. This gel based ionic skin operated using three modes, including capacitive, resistive and triboelectric sensing modes, which can be easily switched simultaneously within one device using a simple sandwiched structure design, as shown in Figure 5B. In addition, the poly (ionic liquid) skin can be easily integrated on an effector, such as a gripper, to perceive the shape of objects.

A breathable and biodegradable material has been created for TENG-based e-skins to improve comfortability of wearable e-skins. Peng et al. (2020) developed a breathable, biodegradable, and antibacterial e-skin based on an all-nanofiber TENG with a micro-to-nano hierarchical porous structure, as shown in Figure 5C. The e-skin was fabricated using a sandwiched structure that consisted of three functional layers: an upper polylactic-co-glycolicacid (PLGA) layer for contact electrification, a middle Ag nanowire layer to act as a conducting electrode and antibacterial agent, and a lower polyvinyl alcohol (PVA) layer to act as a flexible substrate and skin contact. The antibacterial and biodegradable capability of the e-skin could be tuned through adjusting the concentration of Ag nanowires and the selection of PVA and PLGA, respectively. The e-skin was demonstrated by detecting whole-body physiological signals and joint movements. The potential impact of humidity, such as human sweat, when using this biodegradable e-skin and environmental pollutants are less clear and worthy of further investigation.

Waterproof TENG-based e-skins can also provide self-powered operation by harvesting water wave energy. A water-proofing liquid-solid contact electrification-based nanogenerator that enables water wave energy harvesting and subtle motion monitoring in water was developed by Liang et al. (2020). The nanogenerator achieved excellent stretchability with a tensile strain of ~200%, and an output performance with an open-circuit voltage of 120 V and a short-circuit current of 18 μA. The e-skin was ultrathin with a thickness of 300 *μ*m and exhibited good durability of 100,000 submerging-emerging cycles. The device was attached on human finger as a stretchable and wearable sensor for sensing finger motion in water, where its ultrathin and stretchable properties make it promising for use as a self-powered e-skin for soft robotic systems.

Highly sensitive pressure measurements are needed for soft robotic systems, especially when dealing with fragile objects. Chen et al. (2018b) developed a multifunctional e-skin system that integrated the sliding sensing, pressure sensing and power supply elements. This e-skin system consisted of three layers that functioned for sliding detection, pressure detection, and energy supply. A fingerprint inspired TENG, that consisted of four spiral electrodes, was proposed to detect both sliding direction and speed by monitoring the change in voltage. The pressure sensing and power supply measurements were achieved using a conductive elastomer with a hybrid porous microstructure to provide piezoresistance and act as a porous supercapacitor. This type of e-skin has potential to be used to detect complex objects using highly sensitive pressure and sliding measurement, which is suitable for wearable devices and integrating with soft robots.

A bioinspired self-powered TENG-based e-skin was developed by Yao et al. (2020), which mimicked the surface morphology of natural plants. Interlocking microstructures were generated on tribo-layers to enhance triboelectric effects. By using polytetrafluorethylene (PTFE) with fine-scale burrs on the microstructured triboelectric-surface, the sensitivity for pressure measurement was increased by 14-fold. The ultra-sensitive e-skin was attached to a robotic hand, as shown in Figure 5D, for tactile sensing and texture object recognition. The e-skin can be also used to discriminate objects of different hardness. A multifunctional and integrated sliding sensor with high pressure sensitivity and stretchability (200%) was also developed by Yuan et al. (2020). The e-skin combined a capacitive sensing element with a TENG-based sensor and an air gap introduced in the capacitive part significantly increased the sensitivity to applied pressure. The TENG element was able to detect the sliding displacement and velocity of the contact object, while the capacitive section was able to measure pressure. By integrating the two sections together, the thin and flexible skin could be formed and used for sensing a variety of motions such as gripping, holding and sliding of a gripper. This highly sensitive e-skin is of interest for soft robotic manipulators and intelligent recognition for effective avoidance of unexpected slipping and damage during the gripping process.

### TENG-Based Soft Actuators

**Figure 6:**
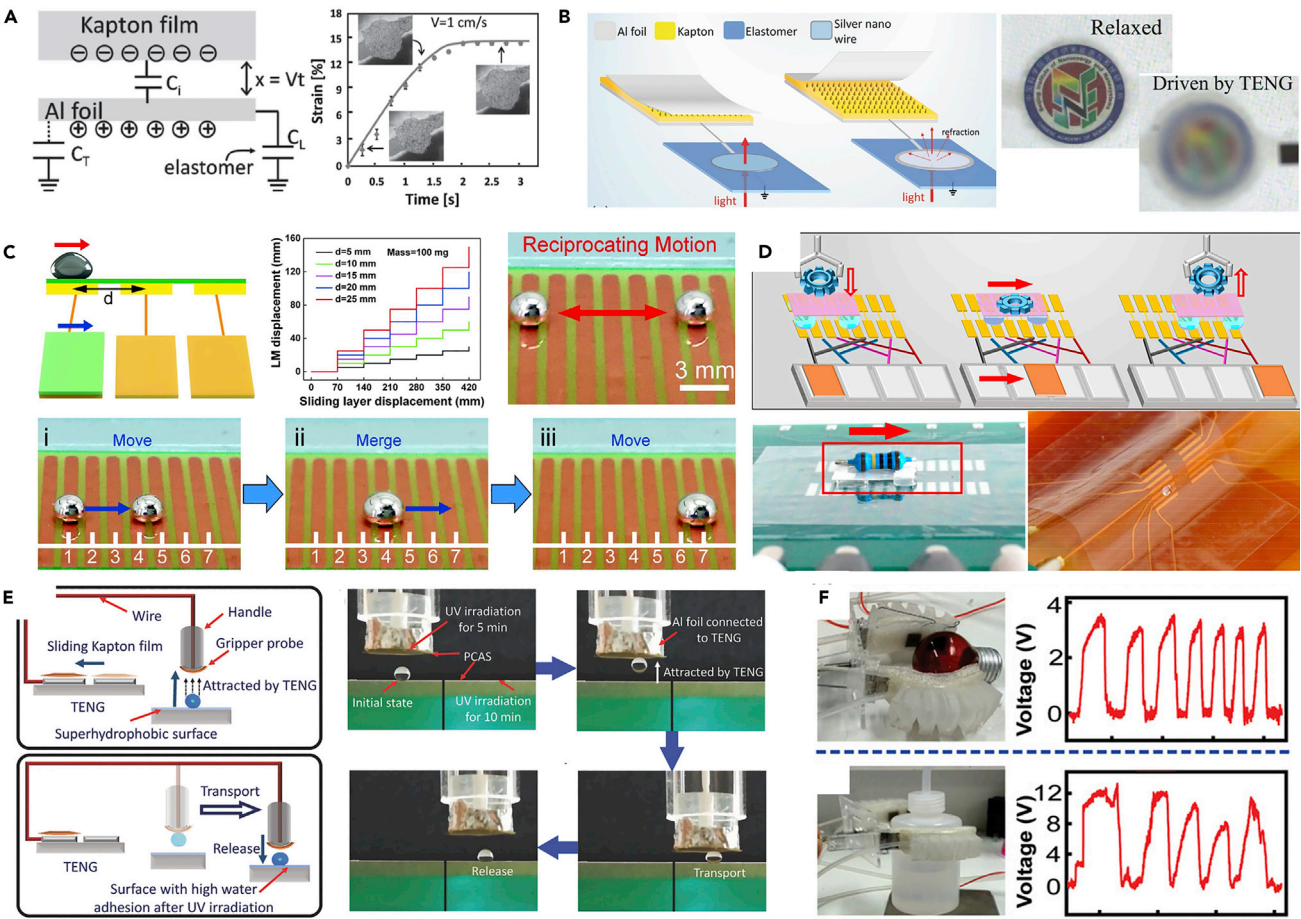
TENG-based Actuators and Actuation Systems (A).integrated TENG-based DEA system. (B) triboelectric tuneable smart optical modulator (SOM), which can change light transmission through the elastomer using refraction. (C) triboelectric effect-driven liquid metal actuator (TLMA) for reciprocating and merging of liquid metals (LMs). (D) self-powered microfluidic transport system based on TENGs. (E) smart microfluidic system based on TENGs and a photo-controllable adhesion surface (PCAS) for long distance transport of micro-/nano-droplets and precise patterning of microfluidics. (F) soft-rigid hybrid actuator based on a conductive sponge/porous silicone-based TENG (SS-TENG).

TENGs can be integrated into soft actuators and used as an autonomous power supply and operate as a self-powered sensor. Actuators integrated with TENG-based sensors or power supplies have been explored by researchers in recent years. As a power supply, a flexible TENG provides the advantages of large output power, low cost, easy fabrication and high conversation efficiency. These characteristics offer great potential for TENGs to be integrated into soft actuators. By integrating a TENG with a thin film dielectric elastomer actuator (DEA), the DEA can be directly powered and controlled by the output of the TENG, which demonstrates a self-powered actuation system for a variety of practical applications in the fields of electronic skins and soft robotics. Chen et al. (2016) used a single-electrode TENG that was integrated with a thin film DEA to form an actuation system, as shown in Figure 6A. The TENG operating in the contact-separation mode and consisted of Kapton PI film and aluminum foil. A TENG with a surface area of 100 cm^2^ could induce a strain of 14.5% in the DEA which had an electrode diameter of 0.6 cm. Using a slow velocity of 1cm/s, the transient deformation and the strain of the dielectric actuator could be controlled by the separation motion of the TENG. The results demonstrated the compatibility between the TENG and DEA mechanisms, which provides a promising method of using self-powered TENG to drive DEA devices. To predict the dynamic response of the TENG-based dielectric actuators, the team developed a model which could simulate the influence of both the viscoelasticity and current leakage of the device on the output performance of the TENG. A constitutive model for the elastomer film was employed to analyze the DEA's viscoelastic relaxation, which provides a tool to analyze the interaction between the TENG and DEA to design TENG-based dielectric actuators (Chen et al., 2017b). Based on this new TENG-DEA system, the team developed a triboelectric tuneable smart optical modulator, which was able to change light transmission through the elastomer using refraction effects (Chen et al., 2017c). Under the activation of the TENG, the Maxwell force induced by the electric fields was able to manipulate the silver nanowire electrode to generate nanowrinkles, which resulted in the control of the optical transmittance through the elastomer film. The separation motion of an aluminum foil, with an area of 100 cm^2^, could decrease the transmittance from 72% to 40% for the optical modulator, which is sufficient for hiding image information behind the device, as seen in Figure 6B. This device can further promote practical applications in the field of soft MEMS/NEMS, human-robot interaction, and internet of things. A TENG-DEA combined system for a motion-modulated tuneable optical grating (TOGs) was also developed by Chen et al. (2017d). In the TENG-DEA, the TENG was used as the power supply and driving signal for DEAs to control the deformation of the grating array.

Zheng et al. (2017) developed two kinds of self-powered electrostatic actuation systems (EAS) that were integrated with TENGs for manipulating microfluids and fine scale solid objects. The TENG provided the control signal and power source for electrification, while the electrostatic actuators generated the Coulomb force to drive microfluids or small sold objects. Bu et al. (2019) developed a triboelectric effect-driven liquid metal actuator (TLMA), in which the liquid metal (LM) was driven for continuous linear and circular motion and was accurately controlled by the TENG. The reciprocation and merging of the liquid metal and a transport system based on TLMA were demonstrated, as shown in Figure 6C. TENGs were also used as power supply and control switches in a self-powered microfluidic transport system developed by Nie et al. (2018), as shown in Figure 6D. In the system, an electrowetting technique was used for a curved surface where the microfluid can move, while a freestanding TENG provides both driving power and control signal to drive the microfluid. Using the same working principle, the team combined TENGs with a photocontrollable adhesion surface to realize a smart microfluidic system (Nie et al., 2019), as seen in Figure 6E. The TENGs provided the power supply and control signal of the microfluid and the photocontrollable adhesion surface was used as a stop with the assistance of UV light to achieve long distance transport of micro-/nano-droplets. Chen et al. (2019) developed a conductive sponge/porous silicone-based TENG (SS-TENG) that acted as a tactile sensor for a soft-rigid hybrid actuator. When the objects were touched, electric signals are generated from the devices which are able to recognize different objects and materials, as shown in Figure 6F. This SS-TENG-based hybrid actuator has potential as a soft gripper by combining two or three actuators with an interface.

To achieve fast prototyping, 3D printing was introduced to simplify the fabrication of TENG-based actuators. For example, a soft robotic finger with a single-electrode triboelectric curvature sensor (S-TECS) fabricated by multi-material 3D printing was developed by Zhu et al. (2020). One active layer of the devices was directly printed on the upper surface of the finger body by multi-material 3D printing, while the other active PDMS layer was combined with a stretchable electrode layer attached on top of the first active layer. The printed sensor was able to detect a finger curvature of up to 8.2 m^−1^ at a working frequency of 0.06 Hz. This multi-material 3D printing technique provides a novel method to effectively directly print soft robotics and its functional sensor, paving the way to develop sensor-rich soft robotics for real-world applications.

### TENG-Based End Effectors

**Figure 7:**
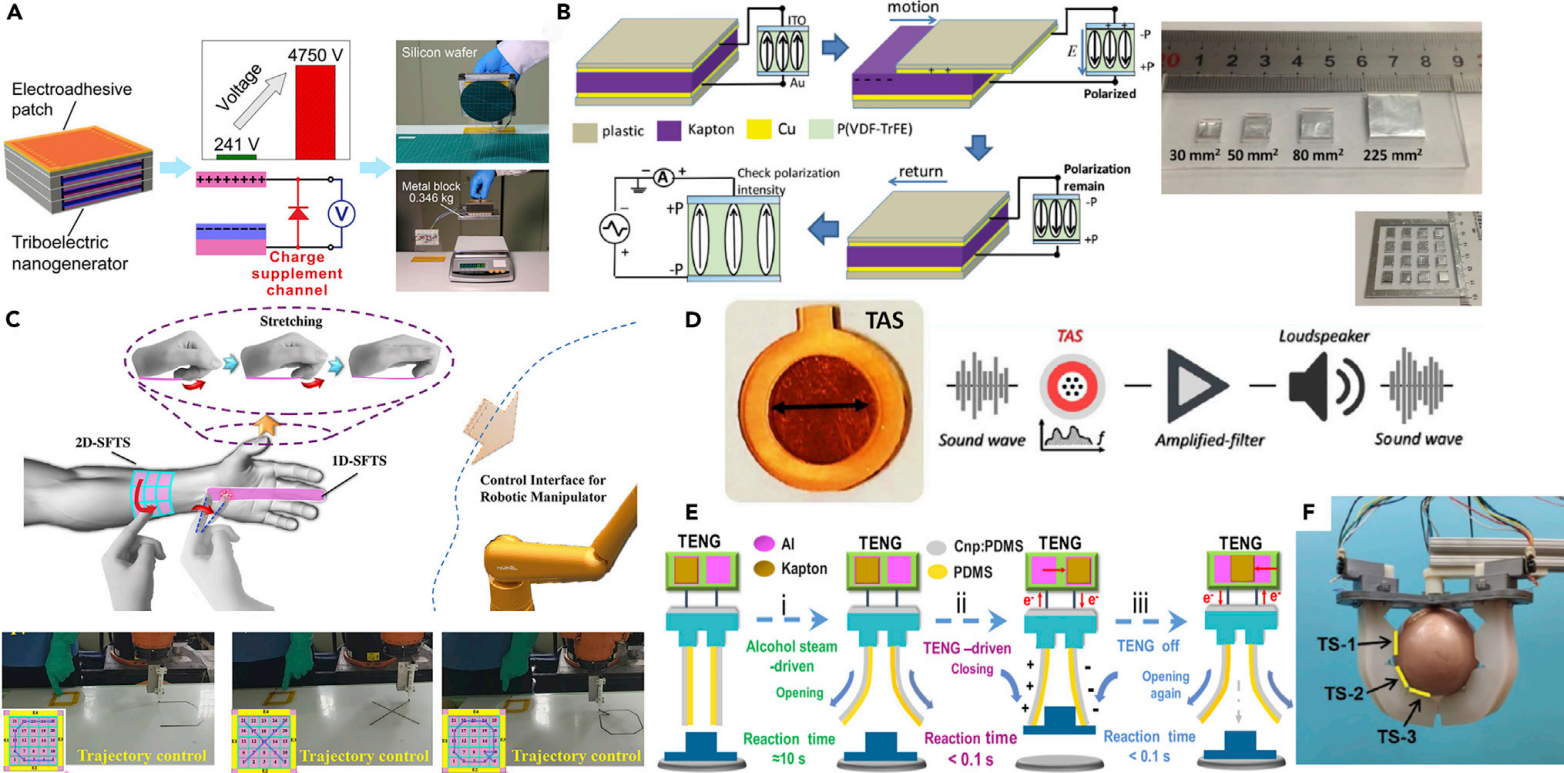
TENG-based Interfaces and Effectors (A) a self-powered electro-adhesion system for grabbing applications. (B) self-powered trace memory system for memorizing motion of 1D and 2D spaces, and displacement current measurement results of P(VDF-TrFE) sample connected to electrodes with different sizes. (C) TENG-based 3D motion sensing system for robotic manipulators. (D) TENG-based auditory interface TAS for hearing aid. (E) TENG-based gripper driven by both ethanol vapor and electrostatic force. (F) smart soft gripper consisting of three TENG-based actuators, grasping an object.

We have seen that TENGs exhibit capabilities for sensing, energy harvesting, and actuation. These characteristics have allowed TENGs to used successfully used as sensors, e-skins, power supplies, and control interfaces for *end effectors*; these are defined as devices that can be installed or attached to a robotic wrist or mounting plate to enable robots to perform their intended tasks. Examples of end effectors include manipulators, grippers, and robotic hands. Xu et al. (2018) applied enhanced TENGs as a power source for a self-powered electro-adhesion system that functioned as a soft gripper. The output of the TENGs was enhanced by a charge supplement channel which maintained an optimal charge distribution throughout the TENG electrodes by use of a replenishing mechanism for dissipated charge. The open circuit voltage of a single TENG unit could be significantly increased by over 10 times, from ~230 V to more than 3300 V, providing a sufficiently high voltage for an electro-adhesive patch to generate sufficient adhesion for practical application. By bonding and combining three layers of the TENG and the electro-adhesive patch together, a self-powered electro-adhesive gripper was fabricated which was able to lift up a metal block of 0.35kg, as seen in Figure 7A.

Integrating TENG-based sensors and e-skins for effectors is attracting increasing research interest in the development of intelligent robotic systems. TENG-based interfaces that enable the control of robotic manipulators were developed by Chen et al. (2015). They developed a self-powered memory system for memorizing the motion in 1D and 2D spaces by coupling a thin film of ferroelectric polymer with a sliding TENG and a single-electrode TENG matrix, as shown in Figure 7B. Electrodes with different sizes, and the TENG matrix with 4 × 4 pixels with a size of 6 mm × 6 mm are also shown in Figure 5B. The memory system was able to record the detailed displacement distance of a sliding TENG and retrieve the motion trace on the surface of the single-electrode TENG matrix. The ferroelectric thin film with a size of 3.1 mm^2^ could record a minimum area change of 30 mm^2^. This self-powered memory systems can be used as an interface to control robotic hands and was successfully demonstrated by Chen et al. (2018c). They developed a self-powered, flexible, triboelectric sensor (SFTS), and creatively combined the 2D-SFTS with the 1D-SFTS to acquire the three-dimensional (3D) spatial information. For a 2D-SFTS, the measurement of current and voltage output of the grids enabled the trajectory and movement in an XY plane to be recorded and the speed and acceleration of the motion can be derived, while a 1D-SFTS was able to track the trajectory in Z plane. The generated 3D trajectory was applied to control the 3D motion of a robotic manipulator and provide real-time control of a robotic manipulator, see Figure 7C. However, the resolution of the motion control is limited to 5 mm since it is determined by the grid divisions. Pu et al. (2018) developed a TENG-based interface for robotic hand control by using a joint motion triboelectric quantization sensor that was attached to human fingers. Synchronous control of a robotic hand was demonstrated using a real-time human-machine interface. The sensor could directly measure a joint's flexion-extension degree/speed by exploiting a high sensitivity of the TENG.

Li et al. (2019) developed a TENG-based tactile sensor by coating a Cu bar onto a PDMS substrate and wrapping the Cu bar in a negative friction silicone rubber material (ecoflex-0030, Smooth on). The sensor was integrated in a gripper to allow it to perceive the shape of a variety of objects by using point-to-point contact sensing. Inspired by caterpillars, Yuan et al. (2019) designed a soft robot that integrated a TENG-based curvature sensor on its back to detect if the back of the robot had touched an obstacle. The triboelectric tactile sensor was fabricated using silicone rubber and liquid metal, and consisted of three layers, including a microchannel layer, a liquid metal layer and the deformation concentration layer. Using the tactile sensor, the soft robot could adaptively crawl across channels with different heights to measure the height of channel.

TENG-based self-powered sensors have been used for sound signal measurement to act as an auditory interface for a hearing aid. Guo et al. (2018) designed a self-powered triboelectric auditory sensor, which was used to construct an electronic auditory system and an architecture for an external hearing aid for intelligent robots, see Figure 7D. The circular-type device consisted of a fluorinated ethylene propylene-covered upper electrode with hole channels, a gap-created spacer, and a Kapton PI membrane connected to a bottom electrode. The outer edge of the PI membrane was fixed by annular acrylic sheet, and the inner film was free in order to vibrate. This low-cost, energy-efficient, and high-fidelity auditory platform provides a promising method for social robotic interaction by measuring sound vibrations. In contrast to contact sensors, Wang et al. (2020a) developed a TENG-based bionic-antennae-array sensor to identify non-contact motion. The non-contact sensor was fabricated by sewing conductive fibers on the surface of the triboelectric dielectric film. The electrostatic balance between the fibers and dielectric film was exploited to detect the non-contact motion. With its high sensitivity, the maximum detected non-contact distance can reach 180 mm, with a displacement resolution of 1 mm. The maximum sensitivity was approximately 5.6 V/mm when the sensor approaches at an angle of 90° and the sensor was attached to a robotic hand to form an alarm system for motion detection.

Yang et al. (2019) developed a soft actuator which was driven by an ionic polymer-metal composite (IPMC)-based TENG, where the deformation of the IPMC film was regulated by the output voltage of the TENG. The triboelectric materials employed were PI and Al foil. A freestanding mode TENG was employed to generate a pulse current signal. The output force of this IPMC-TENG system was 29.6 mN, which can be used to manipulate a variety of small objects. The TENG-based IPMC actuators have application potential in the fields of soft robotics, artificial muscle, and biomimetic devices.

The use of TENG-based vapor-driven actuator materials as effectors offer the advantages of easy fabrication, low cost, and high stability. They have been demonstrated in soft grippers, as seen in Figure 7E. Zheng et al. (2019) modified PDMS to create a vapor-driven actuation material using a simple UV treatment. They combined a vapor-responsive PDMS and TENG to develop two types of dual stimulus flexible actuators and grippers driven by ethanol vapor and an electrostatic force. When the UV/O3-irradiated PDMS film was immersed in an ethanol vapor atmosphere, the treated material gradually undergoes a curling deformation. Another TENG-based gripper has been developed by Chen et al. (2020b), where they presented a finger-like soft actuator with fast response, accurate control, and self-powered pressure and bending sensing capabilities. The soft actuator was integrated with a compliant TENG with micro-pyramid structures and three TENG-based actuators were used to construct a smart gripper. A rubber-based tribo-skin patch with a single-electrode mode was attached to measure contact pressure, and two TENG strips working in contact-separation mode were used to detect bending degrees, as shown in Figure 7F. The gripper could actively detect proximity, contact, and pressure via the self-generated electrical signal. It was noticed that the sensing performance was affected by other factors such as humidity, temperature, and the contact area of the tribo-skin, which varies during grasping, leading to additional calibration challenges.

## PENGs for Soft Robotic Systems and Wearable Devices

**Figure 8:**
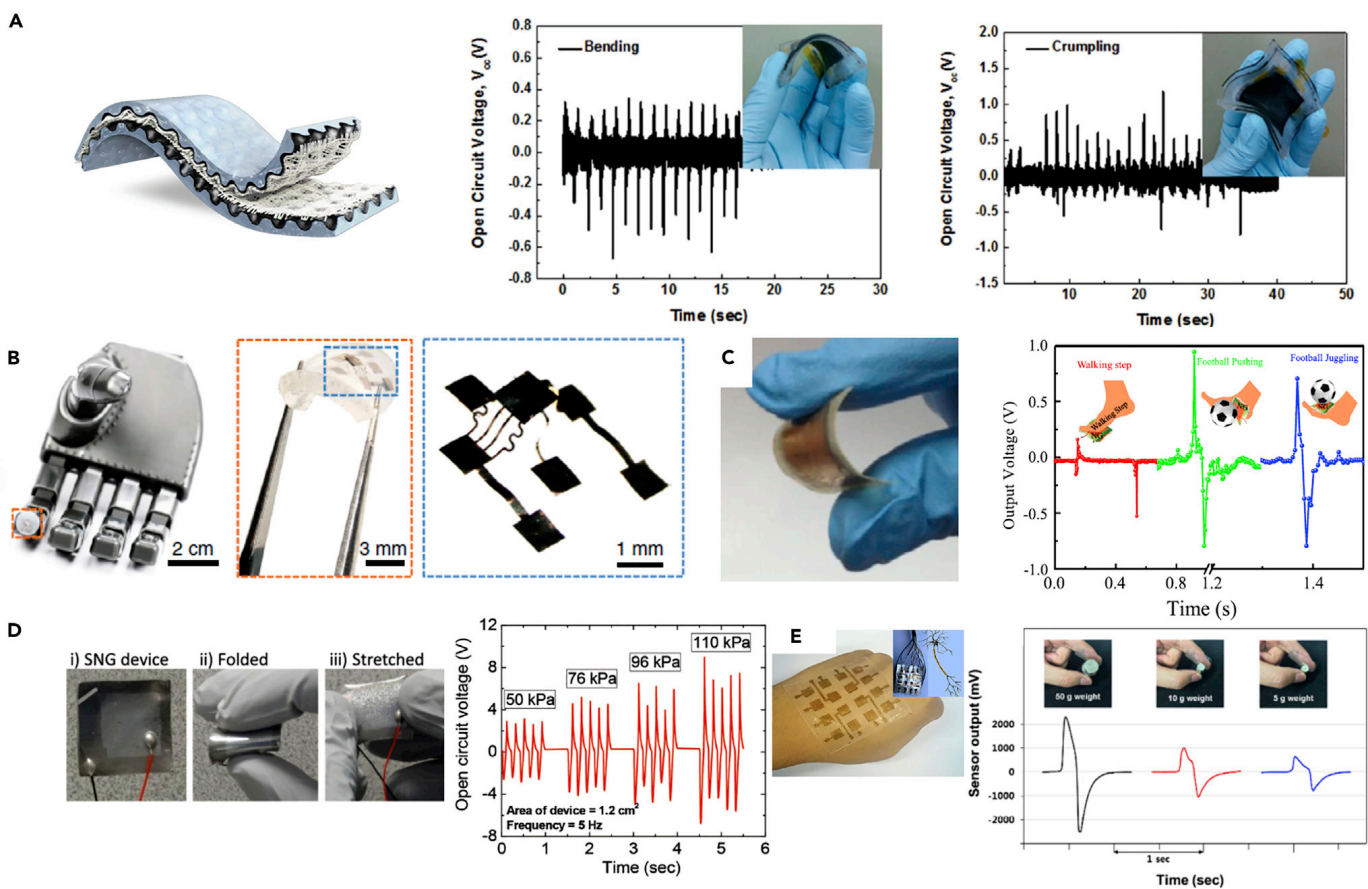
PENG-based Soft Robotic Systems and Wearable Devices (A) open circuit voltage of a graphite-electrode-based PENG for bending and crumpling. (B) 3D piezoelectric microsystem implanted in a robot hand. (C) bio-piezoelectric nanogenerator (BPNG) fabricated using fish swim bladder, which can be used as a self-powered biomedical sensor for wearable electronics. (D) cost-effective PENG-based e-skin which can be applied as a wearable device; relationship of the output circuit voltage with pressure. (E) multi-sensing single-electrode PENG-based e-skin based on the electrospun PVDF nanofibers that can realize steady-state pressure and cold/heat sensing.

Researchers have developed PENGs with good sensing performance and high mechanical flexibility and applied them to soft robotic systems and smart wearable devices. An efficient, omni-directional, and stretchable graphite-electrode-based PENG was developed by Siddiqui et al. (2018) using a 3D micropatterned stretchable substrate and a stacked mat of piezoelectric nanofibers. The PENG was fabricated by combining a stacked mat of electrospun piezoelectric nanofibers with a stretchable graphite electrode on a 3D micropatterned PDMS stretchable substrate, which has a continuous arrangement of valleys and mountains in a curvature, see Figure 8A. The stacked mat of free-standing piezoelectric nanofibers consisted of a nanocomposite of self-poled electrospun nanofibers of barium titanate nanoparticles/polyurethane (BT NPs-PU) and poly (vinylidene fluoride-trifluoroethylene) (P(VDF-TrFE)). The nanofiber PENG exhibits a high stretchability of 40% and a high mechanical durability up to 9,000 stretching cycles at 30% strain. The PENG is cost-effective, environmentally friendly, and can be used as a flexible sensor for soft robotic systems.

Han et al. (2019) extended piezoelectric microsystems to three dimensions in a collection of device architectures that were configured for energy harvesting, robotic prosthetic interfaces, and biomedical implants. This 3D piezoelectric microsystem included a layer of piezoelectric polymer polyvinylidene fluoride (PVDF) and layers of metal (Cr/Au) as electrodes on the upper and lower surfaces. During fabrication, a ribbon of PI (12.5 *μ*m in thickness) served as an underlying support layer for a thin structure of PVDF (9 *μ*m in thickness) in a low-stiffness serpentine shape. During the assembly process, the PI ribbon, bonded at its two ends to an elastomer substrate (700 *μ*m in thickness, prestrain of 10%), buckles upwards, imparting forces onto the back side of the PVDF serpentine to induce an out-of-plane motion, resulting in 3D shape. This 3D piezoelectric microsystem was implanted in a robot hand, as shown in Figure 8B.

By directly using a fish swim bladder (*Catla Catla*, a fresh sweet water fish), Ghosh et al. (2016) developed an efficient bio-PENG fabricated using the fish swim bladder. The device consists of highly aligned natural collagen nano-fibrils, which achieved an open-circuit voltage of 10V and a short-circuit current of 51nA. The instantaneous piezoelectric power generated by the nanogenerator reached 4.15 μW/cm^2^ and an energy conversion efficiency of approximately 0.3%. The device exhibited a linear relationship between the voltage and applied stress so that is can be used as a self-powered biomedical sensor for smart wearable electronics, see Figure 8C. Dahiya et al. (2018) reported on a cost-effective and industrially scalable process for fabricating a robust nanocomposite-based stretchable nanogenerator based on a PDMS substrate. Fabrication of the device was realized by encapsulating the ZnO nanowires (inorganic) in a parylene C polymer (organic) matrix. A high open-circuit voltage of approximately 10 V, a short-circuit current density of approximately 0.11 μA cm^−2^, and a peak power about 3 *μ*W under a vertical compressive force was achieved. The stretchable nanogenerator was demonstrated as a highly sensitive sensor to be worn on fingers, as seen in Figure 8D. Zhang et al. (2020a) developed a flexible PENG-based sensor with a three-dimensional piezoelectric CdS nanowall array structure. Two layers of conductive tape were used to sandwich the CdS nanowall grown from cadmium foil, and then the sandwiched structure was coated with NiO before encapsulating on a PET base. The maximal open-circuit voltage and short-circuit current of the PENG are 1.2 V and 6 nA, respectively. The sensitivity of the PENG-based pressure sensor was 0.143 V/N and the vertically applied pressure and output voltage have a clear linear correlation. The flexible sensor was attached to a human finger to detect the bending angle and frequency. When the finger is fully extended (0°), there is almost no output signal, while peak to peak voltages of approximately 0.6 V and 1.2 V are obtained with bending degrees of 45° and 120°. Similarly, the sensor can be attached on the wrist or keen to measure human body movement. It can be also used as a reliable power supply for small portable electronics, such as LED lamp.

In order to detect a compressive force and horizontal shear force simultaneously, Chen et al. (2018d) developed a flexible three-axial tactile sensor using an imprinted P(VDF-TrFE) piezoelectric micropillar array as an enhanced sensing layer. The distributed and flexible piezoelectric micropillars are highly sensitive to the applied force and are able to generate voltages in response to a compressive and tensile stress. An elastomeric PDMS bump was designed to act as a stress transmission to enable the sensing array to detect the shear force on bumps. The sensitivities for *X*-, *Y*-, and *z* axes force components were 0.3738 V/N, 0.4146 V/N, and 0.3443 V/N. The sensor has potential for use in advanced robots, wearable electronics, and a variety of human-machine interface interactions.

Lee et al. (2017b) created a sensor that consisted of a pair of nonlinear piezoelectric devices based on biaxially grown-ZnO nanorods to detect the bending deformation in terms of the bending angle and bending radius. Based on the anisotropic shape of the one-dimensional nanomaterial ZnO nanorods, the team developed a one-directional aligned device using a dry rubbing process with a quantified degree of alignment for each device. The voltage peak generated according to the bending radius and angle was experimentally measured, and with a bending radius of 31 mm, the open-circuit peak voltage is approximately 100 mV with a bending angle of 22.5° and 30 mV with a bending angle of 90°. The results show a highly responsive linear voltage peak change with the bending angle. The ability of e-skin to achieve a multi-sensing capability is important for the creation of intelligent robots with tactile sensing. Wang et al. (2018b) reported a multi-sensing single-electrode PENG skin device based on electrospun PVDF nanofibers that can realize steady-state pressure and cold/heat sensing based on a single unit, see Figure 8E. The single electrode configuration introduces steady-state sensing and when a pressure is presented, a voltage output is measured. The piezoelectric voltage detects pressure and is not affected by the changes in sensing area since the voltage is determined by device thickness. Cold/hot sensing was recognized using a pulse signal, in order for it to be easily separated from the pressure signal. The sensor can also be applied to transparent interfaces using ITO glass. The fabrication of this PENG is cost-efficient, and it is suggested that this approach can be used for large-scale PENG-based e-skin manufacturing. Sim et al. (2019) developed a PENG-based e-skin that generates “pain” as a warning signal to sharp “prick” contacts and “hot” sensations. The team used piezoelectric P(VDF-TrFE) as the basis for the sensor structure, as it allows both self-power generation and pressure sensing. By exploiting temperature sensing and signal processing based on the Seebeck effect of the ZnO nanowire, instead of the pyroelectric property, the e-skin can distinguish between a “hot” spot using the Seebeck effect and a surface “prick” using the piezoelectric effect. The system therefore enables the detection of pressure and temperature stimuli.

Three-dimensional printing manufacturing has also been used for fabricating PENGs to enable cost-efficient and fast fabrication. Lee et al. (2017a) developed a skin-conformal flexible sensor in which a 3D free-form elastomeric sheet was integrated with a piezoelectric nanofiber mat. A 3D printed mold based on 3D scanned skin surface geometry was used to produce the PDMS elastomeric sheets. The mold was fabricated by a multi-material 3D printer. An electrospun nanofiber mat was prepared as the piezoelectric active layer and integrated with the 3D elastomeric parts. This PENG-based flexible sensor was able to detect various scales of physical stimuli, such as tactile force and pulses. Zhou et al. (2020) also used 3D printing manufacturing to develop a stretchable PENG with a kirigami structure. The PENG was fabricated using piezoelectric BaTiO_3_ NPs in a P(VDF-TrFE) matrix, and silver flake-based electrode with a 3D printable process. The piezoelectric BaTiO_3_ NP/P(VDF-TrFE) ink was printed on a piece of ITO glass that served as a bottom electrode during the poling process to polarize the material to make it piezoelectric. A bottom electrode was also printed using the same Ag flake/P(VDF-TrFE) conductive ink to achieve the 3D printed PENG. The device was able to be mounted onto wearable textiles, such as a sock to form an energy harvester which harvests foot stamping energy and can be used as a self-powered gait sensor. It is noticed that the joints and electrical connections in this device may become the failure point when subjected to a large stress.

## Applications of Integrated TENG and PENG

Advanced *multifunctional* sensing and self-powered flexible devices have been developed by integrating both TENGs and PENGs. Such hybrid soft systems have shown potential in soft robotic systems and wearable devices to combine the advantages of TENGs and PENGs. In addition, the integration of the two mechanisms helps to eliminate the limitations of using only a single technology. For example, the output voltage and power can be greatly improved due to the combination of piezoelectric and triboelectric effects in Figure 2. In addition, multiple sensing modes from touch and stretch can be realized by integrating TENGs and PENGs.

**Figure 9:**
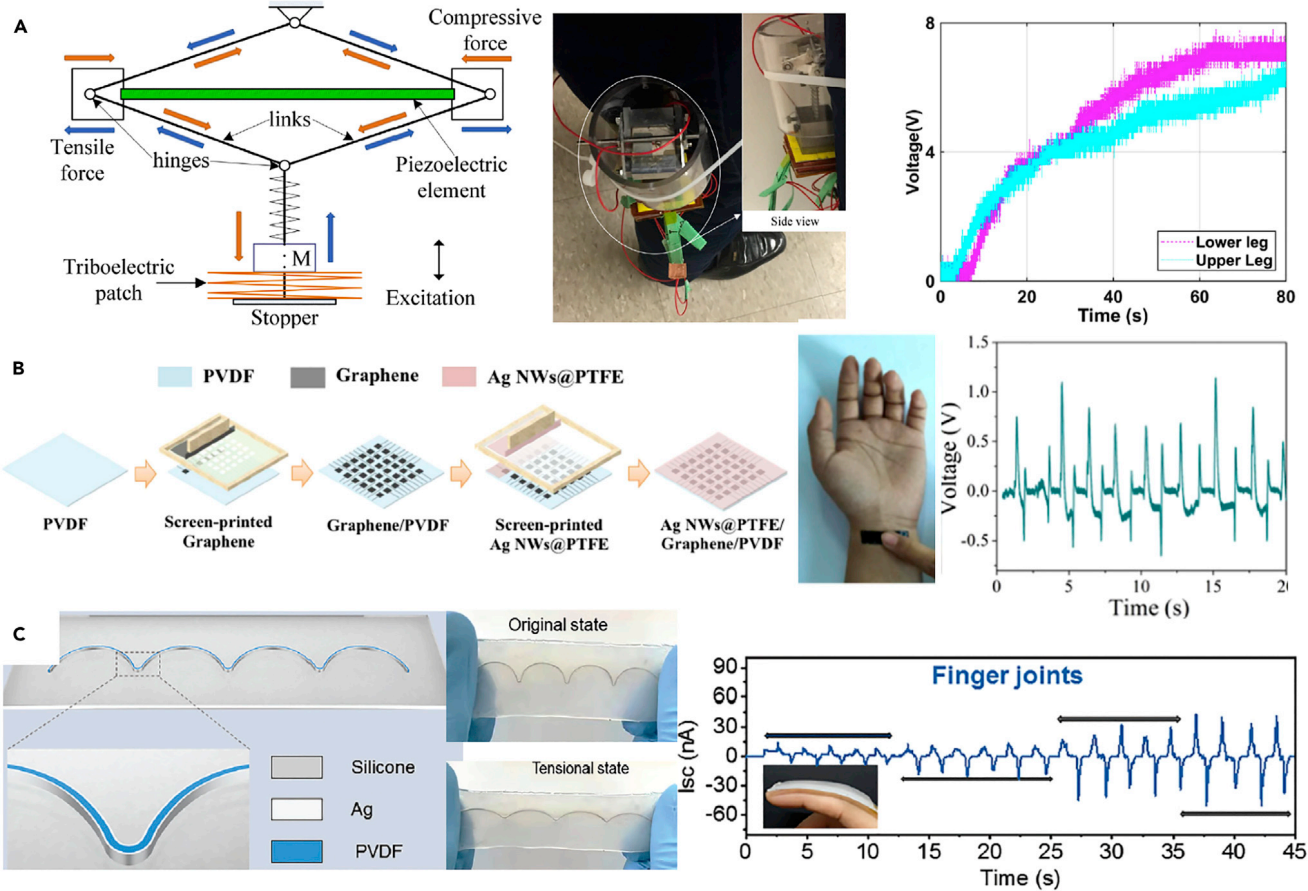
Integrated TENG- and PENG-based Flexible Sensing Devices for Soft Robotic Systems (A) high-power density, high-bandwidth energy generator with a piezoelectric energy harvester (PEH) patch and TENG patch. (B) self-powered flexible antibacterial tactile sensor based on multiple triboelectric-piezoelectric-pyroelectric effects; it can perform multifunctional tactile sensing of pressure and temperature. (C) flexible and stretchable dual mode nanogenerator (FSDM-NG) for human motion sensing and information interaction; a demonstration of finger bending measurement.

Li et al. (2018) developed an energy generator with a piezoelectric energy harvester patch, a TENG patch, a spring-mass system and an amplitude limiter, as shown in Figure 9A. When a base excitation is applied, the spring is deformed, thereby imposing a vertical force to the bottom joint. The force direction is altered to horizontal and the magnitude of the force is amplified. This device shows a high-power density output with a high bandwidth, which can benefit soft robotic applications, especially with an optimized structural stiffness.

A self-powered flexible antibacterial tactile sensor based on triboelectric-piezoelectric-pyroelectric multi-effects has been developed by Ma et al. (2019a), which is utilized to differentiate between external stimuli by analyzing the coupled bimodal voltage signal with a response time difference. The sensor was based on a sandwich structure and consisted of an inner graphene electrode and an outer PTFE and a PVDF film, as shown in Figure 9B. Based on the triboelectric effect of the PTFE and the piezoelectric and pyroelectric effect of the PVDF, the sensor was able to perform multifunctional tactile sensing. The tactile sensor features excellent sensing sensitivity of 0.092 V/kPa and 0.11 V/°C for pressure and temperature, respectively. The sensing and antibacterial properties have been demonstrated by attaching it to a wrist that is touched by a finger, see Figure 9B. A flexible and woven textile device was developed by Kim et al. (2019), where the device provides a variety of features, including capacitive tactile sensing, piezoresistive strain sensing, triboelectric and piezoelectric energy harvesting. The device consisted of a piezoelectric composite, PDMS, CNT, and silver nanowire (Ag NW) layers. The CNT is used as a filler for the piezoelectric composite to improve the piezoelectric properties by using the conductive nanotubes to enhance the internal electric field in the materials. PDMS hemi-spherical structures were formed on the warp functional threads to improve the triboelectric effect, thus providing a large contact area and gradual contact and separation motion. The device was able to detect the tactile force and position by measuring the capacitance and the strain force using resistance, while the electrical power can be generated by the TENG- and PENG-based energy harvesting. The prototype consists of 10 × 10 sensor arrays in a 20 mm × 20 mm area. The maximum power was 108 μW and 60.6 μW for triboelectric and piezoelectric harvesting, respectively. The peak-to-peak open-circuit voltage is approximately 12V for TENG and 18V for PENG with a frequency of 4Hz.

Motion recognition and information interaction sensors with mechanical flexibility and stretchability are key functional modules as interactive media between mechanical motion and electric signals in intelligent robotic and rehabilitation training systems. Fang et al. (2020) developed a piezoelectric-triboelectric hybrid self-powered sensor that can be used to extract energy from biomechanical motions such as blinks and facial muscles. The triboelectric sensor was achieved by contact between Cu and PDMS, while the piezoelectric sensor was made by depositing PVDF fiber arrays on a PCB substrate. The open circuit voltage and short circuit current of hybrid self-powered sensor with a projected area of 30 mm × 25 mm was 1.2 V and 30 nA, respectively. The ultra-thin thickness, high stretchability and superior geometry control features of device makes it promising for e-skins, artificial muscles and soft robotics. The hybrid sensor was integrated on a smart mask for recognizing biomechanical motion and an overall accuracy of 87.9% was achieved. Liu et al. (2020b) developed a flexible and stretchable dual mode nanogenerator for human motion sensing and information interaction, based on the integration of PENG and TENG. In the piezoelectric mode, the hybrid device can effectively monitor the bending angle of joints such as finger, wrist and elbow from 30° to 90°. Under the piezoelectric mechanism, with an increasing linear frequency from 1 to 2.5 Hz, the open-circuit voltage and the short-circuit charge (*Q*) remained almost constant at approximately 16V and 8.8 nC, and the short-circuit current increases from 62 nA to 175 nA; the frequency dependence of current originates from is *I* = *dQ*/*dt*. In the triboelectric mode, text and logic information transfer are encoded using Morse code and logic gates, respectively. With a single electrode TENG, the open-circuit voltage and the short-circuit current are 45 V and 150 nA. By combining these two sensing mechanisms, multiple modes of sensing from touch and stretch can be achieved. The sensor was attached to fingers, wrist, and elbow to detect a bending motion, as seen in Figure 9C, and has potential to be adapted for more complex sensing in the intelligent interaction of robots and rehabilitation training areas.

In order to improve the output performance of nanogenerators, Shi et al. (2019) designed an innovative hybridized nanogenerator combining different mechanical energy harvesting techniques into a single device. By sharing the electrodes, the flexible PENG was coupled with a single-electrode TENG based on a cellulose/BaTiO_3_ nanocomposite airgel paper, which integrated the hybridized nanogenerator into a single device. Due to the combination of piezoelectric and triboelectric effects, the output performance was greatly improved with an output voltage and power of 48 V and 85 μW, respectively. This work provides a novel, easy, and efficient way to increase the output of nanogenerators.

## Conclusions and Perspectives

There are several growing research directions that will benefit significantly from the ability of active materials to intelligent soft robotic and machine systems that use triboelectric- and piezoelectric-based devices, or a combination of both. While significant progress has been made over the last five years, challenges still remain and need to be addressed for soft robotic systems. Much of the work to date in the field has been applied to stretchable and flexible sensors and e-skins, and there are great opportunities for the development of triboelectric- and piezoelectric-based soft actuators and effectors, especially for applications such as soft manipulators, grippers, and microfluid systems.

Other challenges and opportunities for TENG and PENG in soft robotic systems are the integration of e-skin and power supply for soft actuation. Soft robotic systems require fluid flow tubes or electricity wires for sensing and actuation, which introduce new challenges in the development of self-powered untethered robots and wearable devices. TENG and PENG devices provide opportunities to realize self-powered sensing and systems through their unique energy harvesting mechanisms and the ease-of-detecting small voltages and current. However, self-powered actuation is more challenging due to the additional energy demands that can be beyond the power levels that PENG and TENG could provide. It is therefore advantageous to couple nanogenerators to low power actuation mechanisms. Advanced manufacturing technologies, such as 3D printing, which can integrate soft materials, TENG or/and PENG, electronics into soft actuator and robotic systems, provide opportunities to accelerate prototyping and manufacturing the devices. Due to the challenges raised by the complex nonlinear behavior of soft bodies, efficient methods and techniques should be developed for the simulation and control of the TENG-or/and PENG-based soft robotic systems to facilitate their development and optimization. Current state-of-the-art modeling techniques are mainly focused on the aspect of materials design and development.

It is promising to combine TENG and PENG together for soft robotic systems and wearable devices to fully utilize their advantages and eliminate possible limitations. For example, it is possible to couple the TENGs and PENGs to achieve more efficient sensing and energy harvesting for application. TENG-based actuators are still in their infancy and it would be highly useful to understand the factors that can significantly improve actuation force and bandwidth. In addition, limited TENG- or/and PENG-based soft robots and devices in the microscale are reported in literature. We have also seen the advantages of combining TENG and PENG devices with additional materials for multi-functionality, such as optical properties, antibacterial, or self-healing capability. The current state-of-the-art robots and devices are mainly at millimeter or centimeter scale, which is limited to the application in small enclosed and confined spaces, such as blood vascular or intestinal tract for disease diagnostic, biomedical treatment, or drug delivery. The microscale TENG- or/and PENG-based soft robotic systems can therefore be a promising direction in the field. It is expected that with the advances in material development, novel design methodologies, and innovative actuation and propulsion mechanisms, TENG- and/or PENG-based soft robotic systems will be practically use in a broad range of applications in the future.
